# *De novo* grafted coiled-coil peptides as p53/*h*DM2 inhibitors

**DOI:** 10.1039/d6cb00063k

**Published:** 2026-06-11

**Authors:** Freya Spain, Diana Gimenez, Amanda M. Acevedo-Jake, Bram Mylemans, Nikolas J. Brooks, Boguslawa Korona, Danny T. Huang, Thomas A. Edwards, Aneika C. Leney, Laura Itzhaki, Derek N. Woolfson, Andrew J. Wilson

**Affiliations:** a School of Chemistry, University of Birmingham Edgbaston B15 2TT UK a.j.wilson.1@bham.ac.uk; b School of Chemistry, University of Bristol, Cantock's Close Bristol BS8 1TS UK; c Novo Nordisk Foundation Center for Protein Design, Department of Biology and Department of Drug Design, University of Copenhagen Copenhagen Denmark d.n.woolfson@bio.ku.dk; d School of Biosciences, University of Birmingham Edgbaston B15 2TT UK; e Department of Pharmacology, University of Cambridge Cambridge CB2 1PD UK lsi10@cam.ac.uk; f Cancer Research UK Scotland Institute, Garscube Estate, Switchback Road Glasgow G61 1BD UK; g School of Cancer Sciences, University of Glasgow, Garscube Estate, Switchback Road Glasgow G61 1QH UK; h College of Biomedical Sciences, Larkin University 18301 N Miami Ave #1 Miami Florida 33169 USA; i Astbury Centre for Structural Molecular Biology, University of Leeds Woodhouse Lane Leeds LS2 9JT UK; j School of Biochemistry, University of Bristol, Medical Sciences Building, University Walk Bristol BS8 1TD UK; k Max Planck-Bristol Centre for Minimal Biology, University of Bristol, Cantock's Close Bristol BS8 1TS UK; l School of Chemistry, University of Leeds Woodhouse Lane Leeds LS2 9JT UK

## Abstract

Dysregulation of protein–protein interactions (PPIs) plays a key role in disease progression. PPI interfaces have long been considered challenging targets to drug due to their large surface areas and lack of well-defined binding sites suitable for small molecules. Peptide-based ligands offer the opportunity to mimic the action of a native binding partner to inhibit PPIs. Previously, we have used the extensively characterized *de novo*, parallel, homodimeric coiled coil, CC-Di, as a template to design selective inhibitors of the NOXA-B/MCL-1 PPI. To further establish that coiled coils offer the possibility of modulating α helix-mediated PPIs, we show that this approach can be adapted to design coiled coils that are competitive inhibitors of the p53/*h*DM2 PPI with sub-micromolar (μM) affinities, as demonstrated by fluorescence anisotropy.

## Abstract 2

Dysregulation of protein–protein interactions (PPIs) plays a key role in disease progression.

## Graphical abstract

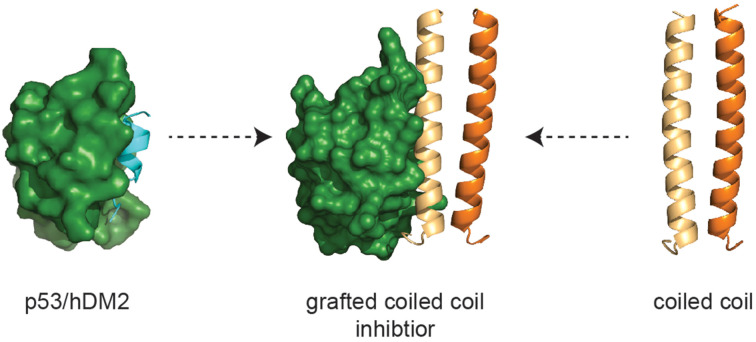

## Introduction

Protein–protein interactions (PPIs) mediate the vast majority of biological processes^1–3^ and can play important roles in disease. The use of synthetic molecules to modulate PPIs is therefore attractive; for instance, they can be used as probes to understand the role of a protein or PPI in a biological process and serve as starting points for drug discovery. The last 10–15 years has seen progress in the development of small molecule, antibody and peptide-based modulators of PPIs for a number of indications.^4–6^ However, developing potent and selective competitive PPI inhibitors remains challenging; this arises due to the large surface area, lack of well-defined pockets and dispersed arrangement of recognition handles that define many PPIs. Peptides are a particularly promising class of PPI inhibitor^6^ owing to the molecular recognition capabilities they offer for high affinity and selective target binding. Moreover, peptides and peptidomimetics can mimic a native binding partner with high fidelity.^6^ For instance, mini-,^7,8^ and designed proteins,^9–13^ stapled peptides,^14–17^ foldamers^18–21^ and grafted proteins^22^ are all suitable design templates for inhibition of α-helix mediated PPIs.

**Fig. 1:**
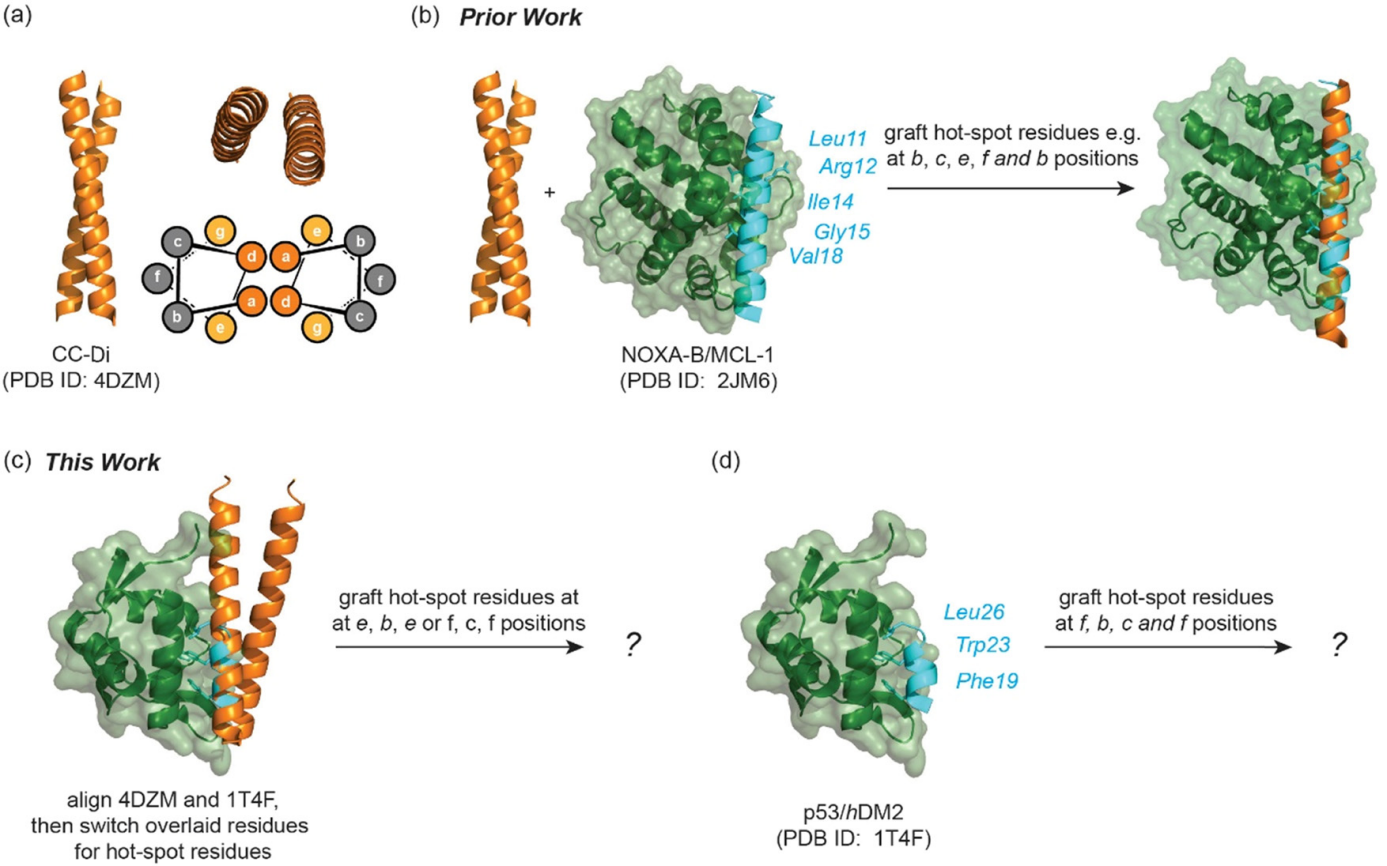
Design of coiled coils as PPI inhibitors: (a) structure of parallel homodimeric coiled coil (CC-Di, PDB ID: 4DZM, orange) characterised by a series of heptad repeats as illustrated. In prior work, (b) hot-spot residues (cyan sticks) from NOXA-B (cyan) have been grafted onto solvent exposed residues of CC-Di to generate NOXA-B/MCL-1 inhibitors (MCL-1 in forest green, PDB ID: 2JM6). In this work; (c) p53 (cyan) and CC-Di (orange) were aligned and residues (shown as sticks) manually selected for grafting to generate candidate p53/*h*DM2 inhibitors; or, (d) hot-spot residues (cyan sticks) from p53 (cyan) were grafted onto similar positions as used previously (*h*DM2 in forest green, PDB ID: 1T4F).

An alternative approach to present a helical binding epitope is within the context of a coiled coil. Coiled-coil peptide assemblies are stable well-understood scaffolds.^23^ Typically, coiled-coil sequences have heptad repeats (abcdefg)_*n*_ where the *a* and *d* positions are often hydrophobic residues that promote the folding and direct association of two or more α-helical peptides (Fig. 1a).^23^ Moreover, the solvent-exposed *b*, *c* and *f* positions can be varied to graft on functional residues, *e.g.* for target binding. Coiled-coil assemblies have been used to rewire & regulate signalling pathways, and, as delivery or therapeutic reagents.^24–27^ Many PPIs are categorised by hot-spot residues (*i.e.* residues that make a significant contribution to the free energy of association – ΔΔ*G* ≥ 4.2 kJ mol^−1^ – as demonstrated by methods such as alanine scanning).^28^ Our groups^29,30^ and others^31,32^ have shown that grafting of hot-spot residues onto the outer surface of coiled coils offers promise as an approach to develop potent PPI inhibitors. Previously, we used parallel homo and heterodimeric coiled coils as templates for the design of potent and selective NOXA-B/MCL-1 inhibitors (Fig. 1a and b);^29^ members of the BCL-2 family that regulate apoptosis^33–35^ and which have received attention as therapeutic targets.^36,37^ Here, we demonstrate the generality and adaptability of this approach through the design of parallel coiled coils that selectively inhibit the p53/*h*DM2 interaction. We chose p53/*h*DM2 because, like NOXA-B/MCL-1, it is a helix mediated interaction that relies on an *i*, *i* + 4 and *i* + 7 constellation of hot spot residues,^38^ whilst overexpression of *h*DM2 in many tumour cells inactivates the tumour suppressor p53, promoting tumour growth, making it a therapeutically important target^39,40^

To develop parallel coiled coils as p53/*h*DM2 inhibitors we selected the *de novo* designed coiled-coil peptide CC-Di (Fig. 1a; PDB: 4DZM)^41^ as a template, based on our success in using it to develop inhibitors of the NOXA-B/MCL-1 interaction. CC-Di has been extensively characterised. It relies upon the *g*, *a*, *d* and *e* sites of the heptad repeat for assembly, leaving the *b*, *c* and *f* positions available for grafting. The scaffold exhibits well-defined oligomerization behaviour (*i.e.* forms only homodimers, not higher order oligomers), unlike variants of the naturally occurring GCN4 leucine zipper that have been exploited previously in protein grafting involving the C-terminal region of HIV-1 gp41, which mediates HIV-1 entry into cells,^42^ and the p53 transactivation domain (p53_15-29_ or p53_TAD_).^32^ The p53_TAD_ recognises *h*DM2 primarily through an *i*, *i* + 4 and *i* + 7 constellation of residues, F19, W23 and L26,^38^ whilst BH3 domains such as NOXA-B also employ *i*, *i* + 4, *i* + 7 and *i* + 11 constellations together with additional residues in recognizing their BCL-2 family (such as MCL-1) targets, resulting in strong binding interactions.^43^

For the first approach, alignment of the crystal structures of CC-Di (PDB ID:4DZM) and p53/*h*DM2 (PDB ID:1T4F) in PyMOL overlayed the key *i*, *i* + 4 and *i* + 7 of the p53_TAD_ in two alternate registries: the *e*, *b* and *e* positions (CC-Di-p53-1-3) or *f*, *c* and *f* positions (CC-Di-p53-4) across two heptads. Although the *e* position plays a role in coiled-coil stability we hypothesized that residue variation may be tolerated, given this was the case in our original MCL-1 study. Therefore, CC-Di-p53-1-4 (Table 1, light orange/brown) were proposed, bearing the key p53 hot-spot residues and a number of further modifications introduced on visual analyses of the aligned structure (CC-Di-p53-2: Phe at *c* in heptad 2; CC-Di-p53-3: Glu at *b* in heptad 1, Phe at *c* in heptad 2; CC-Di-p53-4: Glu at *b* in heptad 4). For the second approach, whilst our MCL-1 binding coiled coils (*e.g.* CC-Di_S) required grafting at *b*, *c*, *e*, *f* and *b* positions across two central heptads to cover *i*, *i* + 4, *i* + 7 and other key residues, a minimal *h*DM2 binding motif only required grafting of the solvent exposed *f*, *b*, *c* and *f* positions across two heptads, to mimic the *i*, *i* + 4 and *i* + 7 positions and one further residue (Table 1, dark orange). Using *b*, *f*, *b*, positions might also have been possible, but this would have placed the L22 mimicking side chain at position *e* closer to the helix–helix interface, which was less desirable than our chosen *f*, *b*, *c* and *f* positions. The first design – CC-Di-p53-5 – contained only the minimal F.LW.L sequence. For the second design – CC-Di-p53-6 – we analysed a number of published p53/*h*DM2 co-crystal structures^44–48^ with BUDE Alanine Scan (BAlaS)^49^ (see Table S1) and implemented the best predicted sequence variations alongside others shown to improve potency (*e.g.* the pMI peptide).^50^ This resulted in the grafted sequence: FL.YW.LL; we also altered the starting register of CC-Di to place this grafted sequence as central as possible.

**Table 1:** Library of designed peptides used in this study (with hot-spot residues bold and underlined, and additional variant residues bold, X = Ac or FAM-Ahx-) NOXA-B (grey) and p53 (cyan) with underlined amino acids signifying hot-spot residues. The canonical parent CC-Di (black) is used to generate hybrid sequences that assemble into parallel homodimeric coiled coils *via* two design strategies; (i) alignment of CC-Di (PDB ID:4DZM) and p53/*h*DM2 (PDB ID:1T4F) structures, swapping coiled coil residues for the overlayed p53 hot-spot residues (light orange); (ii) grafted residues involved in p53/*h*DM2 binding at *f*, *b*, *c* and *f* positions of the heptad as exploited in our previous study (dark orange). (Notes: CC-Di-p53-4 arose from a different alignment which matches the (ii) series and is shown brown)

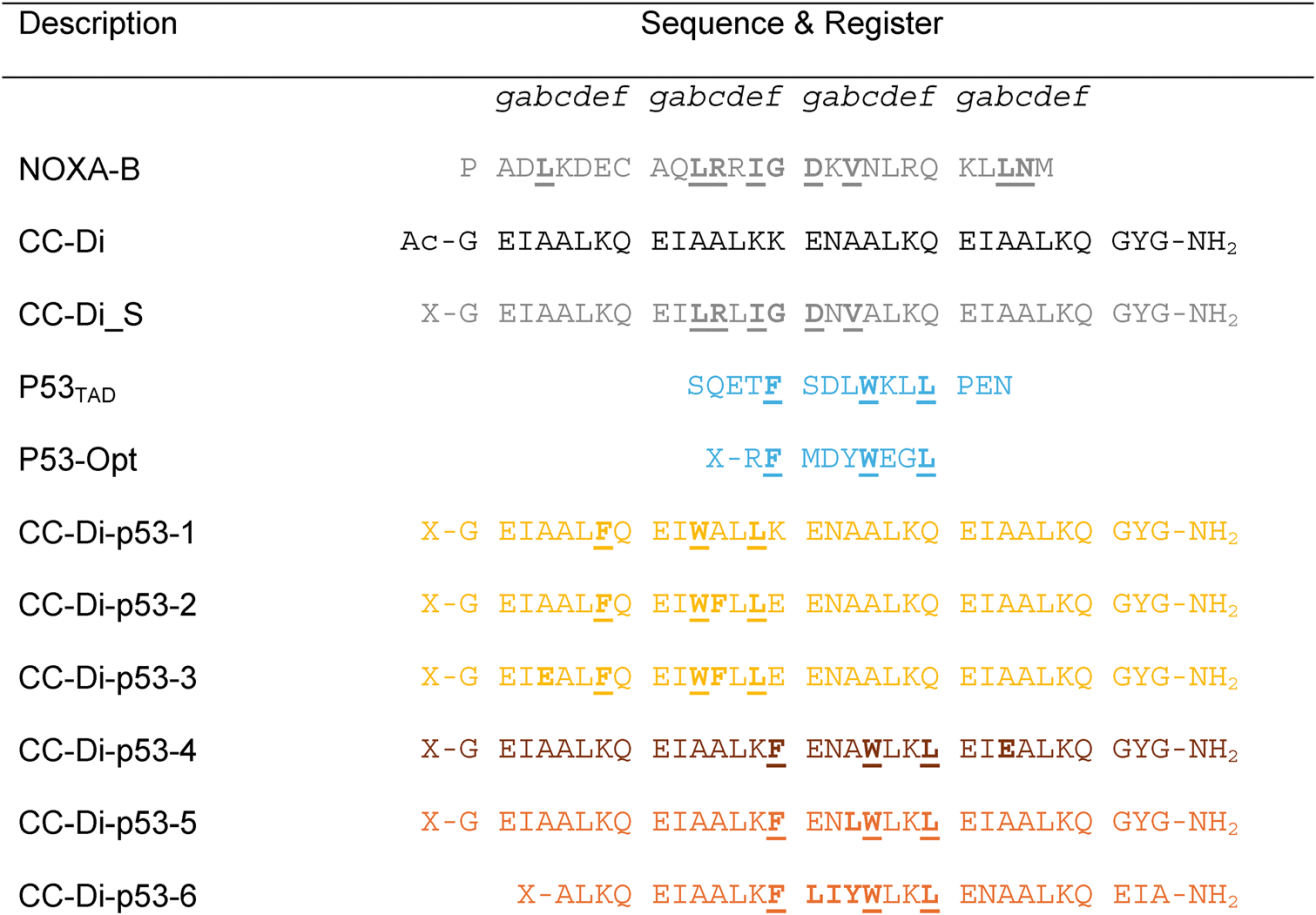

**Fig. 2:**
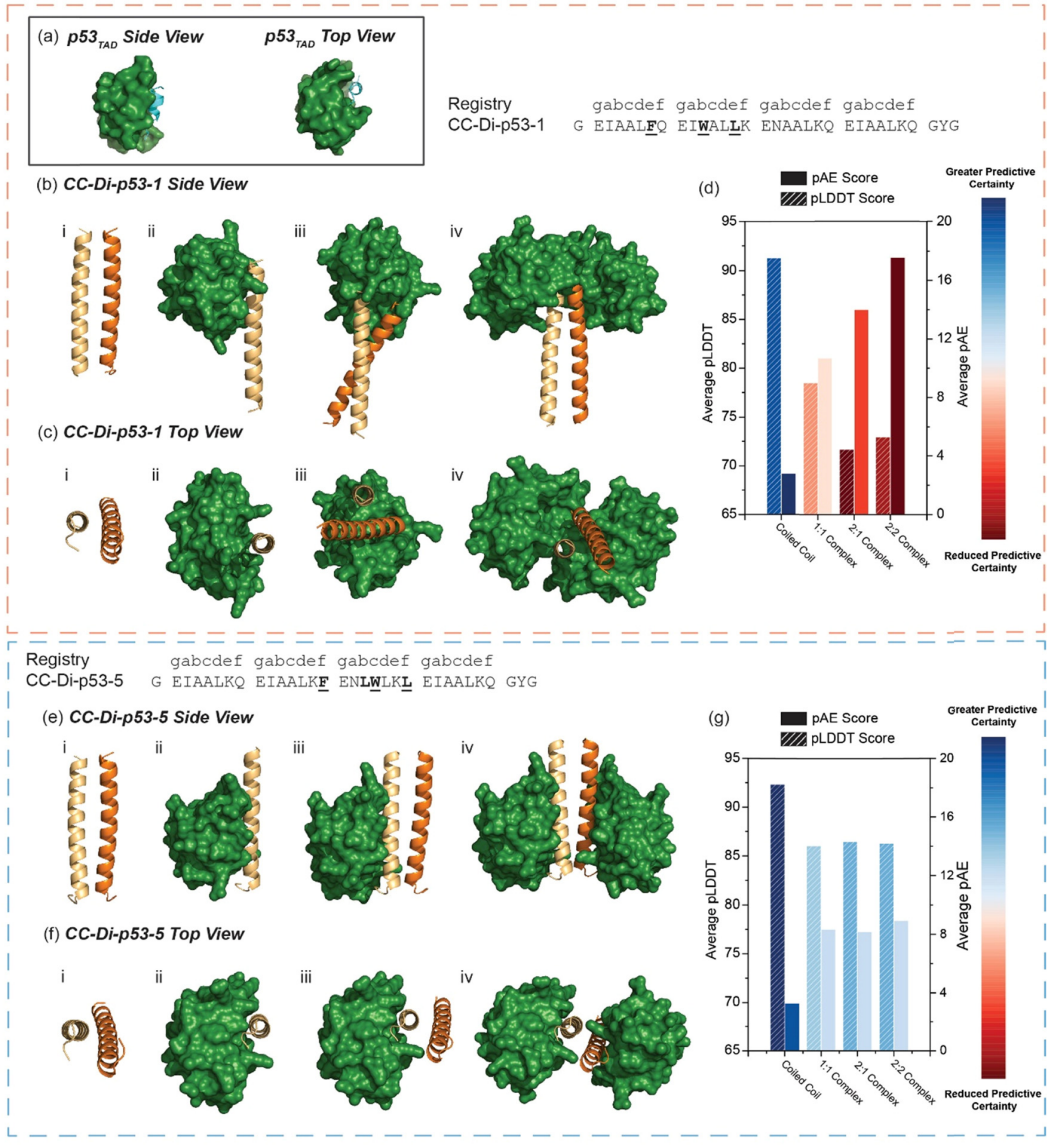
AlphaFold3 models of representative coiled coils CC-Di-p53-1 and CC-Di-p53-5 (yellow and orange) in the absence and presence of *h*DM2: (a) side and top view of the wildtype p53_TAD_/*h*DM2 PPI (PDB ID: 1T4F); (b) side and (c) top view of CC-Di-p53-1 as (i) a coiled coil in the absence of protein, (ii) a 1 : 1 complex, (iii) a 2 : 1 complex and (iv) a 2 : 2 complex with *h*DM2 (forest green); (d) average pAE and pLDDT scores for each CC-Di-p53-1 complex with *h*DM2 (blue indicates greater predictive certainty in the model), where complexes of CC-Di-p53-1 with *h*DM2 become increasingly less likely to form; (e) side and (f) top view of CC-Di-p53-5 as (i) a coiled coil in the absence of protein, (ii) a 1 : 1 complex, (iii) a 2 : 1 complex and (iv) a 2 : 2 complex with *h*DM2; (g) average pAE and pLDDT scores for each CC-Di-p53-5 complex with *h*DM2.

AlphaFold3 was used to predict structures for peptide/*h*DM2 complexes using CC-Di-p53-1 and 5 as representative examples, with pLDDT and pAE scores giving an indication of predictive certainty (*i.e.* confidence) in the models (Fig. 2). The coiled-coil peptides alone gave expected models for parallel, homodimeric coiled coils with low pAE and high pLDDT scores. Hybrid coiled coils where the hot residues were grafted between heptad 1 and 2 were predicted to behave differently compared to those where the recognition site was grafted into heptads 2 and 3 of the coiled coil. The models for CC-Di-p53-1 indicated that neither 2 : 1 nor 2 : 2 complexes (peptide:protein) are likely to be stable, and that the grafted residues failed to reproduce the spatial orientation of the p53 binding residues into the expected sites on *h*DM2 for any oligomer state (1 : 1, 2 : 1 or 2 : 2). It should be highlighted that grafting residues at *e* positions of the heptad (CC-Di-p53-1) removes two lysine residues from the scaffold that are known to support coiled-coil formation.^41^ Modelling of CC-Di-p53-5 suggests that multiple oligomerization states may be observed upon introduction of *h*DM2 including a 2 : 2 complex whereby the designed dimer recruits two copies of protein. In the case of CC-Di-p53-5, the models suggest that the *f*, *c*, *f* placement of hot spots on CC-Di does not hinder dimeric assembly of the peptide when bound to the protein. The models also suggest that the coiled coil effectively recapitulates the spatial orientation of the grafted residues into the expected sites on *h*DM2 where the hot-spot residues bind for all stoichiometries.

**Fig. 3:**
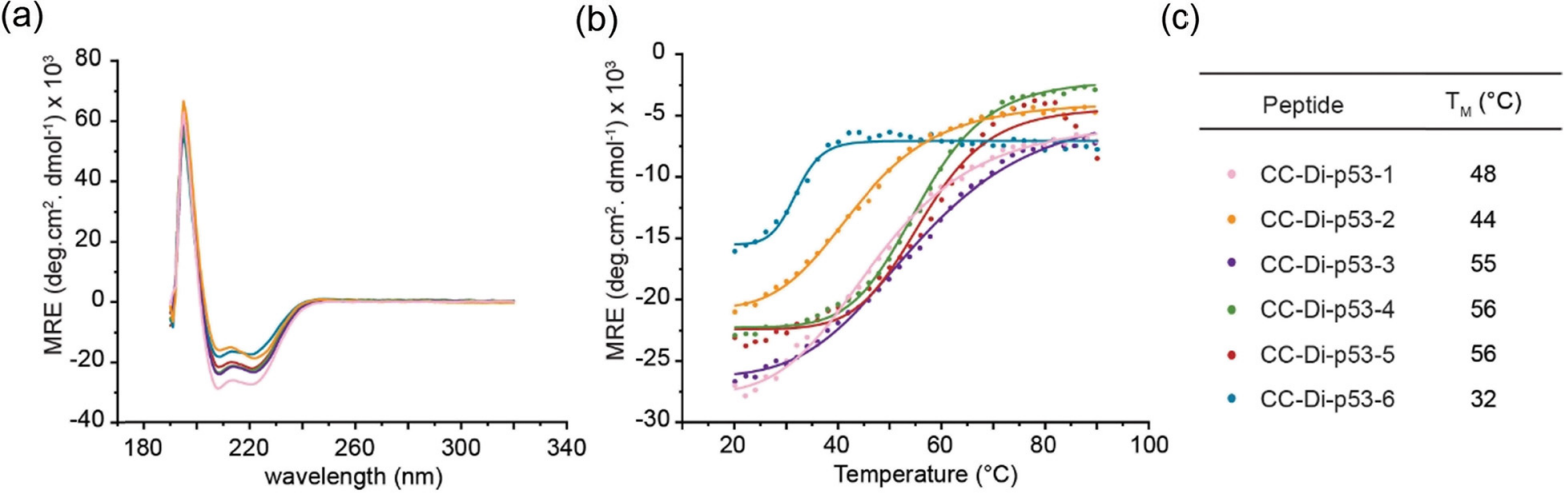
Circular dichroism spectroscopy analyses of p53-coiled coil hybrids: (a) spectral scans of the peptides at 20 °C; (b) thermal denaturation from 20–90 °C, scanning at a wavelength of 222 nm; (c) table of reported *T*_m_ values (experiments were performed at a concentration of 20 µM peptide in PBS pH 7.4 with 30% acetonitrile in a 1 mm cell). The effect of 30% acetonitrile on coiled coil assembly in this case was negligible (Fig. S1).

The designed peptides were prepared using solid phase synthesis bearing 5,6-carboxyfluorescein following an aminohexanoic acid linker or acetyl groups at the N-terminus and C-terminal amidation. Circular dichroism (CD) spectroscopy was used to examine the secondary structure and stability of the peptides in solution. In these experiments, acetonitrile was used as a co-solvent to ensure solubilisation of the peptides; the effect of acetonitrile was found to have minimal impact on secondary structure and reduced thermal stability slightly (see Fig. S1). Hybrid coiled coils that more closely retained the parent scaffold exhibited a high α-helical content with ellipticity comparable to CC-Di (MRE_222_ reported −31.650).^29^ In contrast, CC-Di-p53-5 and CC-Di-p53-6 adopted assemblies with lower helical content, likely due to increased scaffold decoration with residues for recognition of *h*DM2 (Fig. 3a). Thermal denaturation experiments (Fig. 3b) further demonstrated that these sequence modifications influenced coiled-coil stability compared to the parent CC-Di (*T*_m_ reported 75 °C).^29^ Peptides containing more extensive substitutions showed reduced thermal stability, consistent with the incorporation of helix-destabilising residues to enhance target binding (Fig. 3b). Despite CC-Di-p53-1-3 lacking the *e* to *g* salt bridge in the first heptad due to substitution at the *e* position, their melting temperatures were shown to be comparable to those of CC-Di-p53-4 and CC-Di-p53-5. Notably, CC-Di-p53-6 displayed the lowest stability yet had the highest affinity for *h*DM2 by fluorescence anisotropy experiments and native mass spectrometry (see below).

**Fig. 4:**
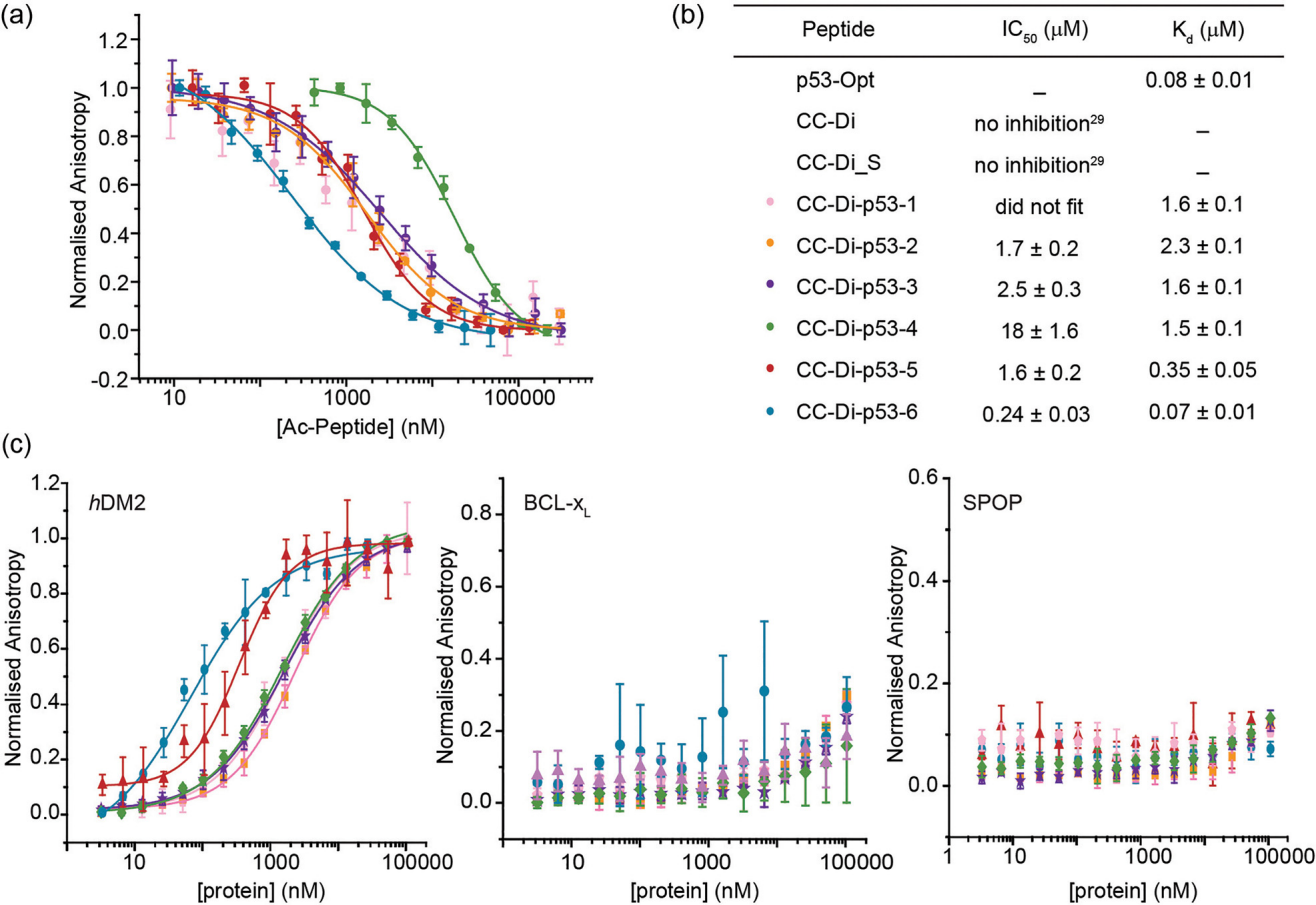
*h*DM2 binding experiments measured by fluorescence anisotropy (FA): (a) fluorescence anisotropy competition assays against the FAM-Ahx-p53-Opt/*h*DM2 interaction (20 mM Tris, 150 mM NaCl, pH 7.6, assays performed in triplicate, peptides acetylated at the N-terminus); (b) table summarising IC_50_ and *K*_d_ values, determined by FA. CC-Di-p53-1 did not fit well to a logistic model and consequently an IC_50_ value could not be determined; (c) variation in peptide tracer anisotropy as protein was titrated in a direct binding assay (*K*_d_ determined using total peptide concentration, titrated against *h*DM2, BCL-x_L_ or SPOP in 20 mM Tris, 150 mM NaCl, pH 7.4, assays performed in triplicate, peptides functionalised with 5,6,carboxy fluoresceine (FAM) and an aminohexanoic acid at the N-terminus).

The ability of the peptides to competitively inhibit the p53/*h*DM2 interaction was evaluated by fluorescence anisotropy competition assays whereby a fluorescently labelled p53 ligand (FAM-Ahx-p53-Opt) was displaced from *h*DM2 using the acetylated coiled-coil p53 mimics (Fig. 4a and b, see Fig. S2 for direct titration of FAM-Ahx-p53-Opt). CC-Di-p53-1 did not fit well to the logistic model for a 1 : 1 competition process suggesting the possibility of a non-specific element to binding and/or tracer interaction, as has been observed previously for p53/*h*DM2 inhibitors.^51^ Peptides CC-Di-p53-2 to 6 showed competitive inhibition in the low or sub-micromolar range. As expected from the sequence design, CC-Di-p53-6 (IC_50_ = 240 ± 3 nM) was the most potent inhibitor, which can be attributed to the more extensive hot-spot grafting onto the scaffold. FAM labelled variants of the coiled coils were tested for direct binding to *h*DM2, and to assess selectivity to a different helix binding target – BCL-x_L_ – and a further non-helix binding protein – SPOP (Fig. 4b and c). All designs bound to *h*DM2 with single digit µM affinity or better; the most potent ligands were CC-Di-p53-5 (*K*_d_ = 0.35 ± 0.05 µM) and CC-Di-p53-6 (*K*_d_ = 0.07 ± 0.01 µM) in concordance with the competition assay and which is comparable in affinity to the tracer peptide (FAM-Ahx-p53-Opt *K*_d_ = 0.08 ± 0.01 µM. This is in the same affinity regime as previously described coiled coils, based on grafted GCN4-derived peptides;^32^ the well-defined assembly behaviour of the CC-Di template used here and the ability to rationally engineer it, may make it advantageous for grafting hot-spot residues. In addition, the peptides did not bind to either of BCL-x_L_ or SPOP, indicating good selectivity for *h*DM2 relative to these two targets. The template CC-Di coiled coil was previously shown to have no inhibitory potency when tested against NOXA-B/MCL-1, BH3/BCL-x_L_, or p53/*h*DM2, whilst CC-Di_S inhibited only NOXA-B/MCL-1, further emphasizing the ability to tune target selectivity for this series of designed coiled coils by choice of grafted residue, which has not yet been demonstrated with GCN4-derived coiled coils.

In our prior study on NOXA-B grafted coiled coils for MCL-1, AlphaFold2 modelling of the complexes suggested that dimeric coiled coils could recruit two copies of MCL-1.^30^ The dimeric coiled coils with high affinity for MCL-1 exhibited a preference for 2 : 2 peptide:MCL-1 ternary complex formation as shown by native mass spectrometry (MS) and analytical ultracentrifugation (AUC). To further probe the nature of *h*DM2 binding for CC-Di-p53-1-6, we attempted AUC and native MS experiments. Sedimentation velocity (SV) AUC on equimolar mixtures of protein and peptide failed to provide interpretable data indicating the complexes may not be stable and/or aggregate under the conditions of the experiment. Consistent with circular dichroism, fluorescence anisotropy experiments and AlphaFold3 predictions, native MS analyses (Fig. S3) revealed minimal complex formation of *h*DM2 with CC-Di-p53-1. The highest affinity interaction observed was between *h*DM2 and CC-Di-p53-6, whereby a 1 : 1 complex dominated the mass spectrum. This is consistent with the high affinity measured by fluorescence anisotropy, and the low coiled-coil stability of CC-Di-p53-6 measured by circular dichroism, suggesting that as we increase grafting on to the scaffold, the coiled coils more readily dissociate to bind to the target. Indeed, it is possible that the low CC-Di-p53-6 coiled-coil stability combined with the reduction in hydrophobic interaction strength during gas-phase analysis within the mass spectrometer, prevented the observance of any 2 : 2 complexes involving CC-Di-p53-6.

Overall, native MS data provides additional evidence of binding between CC-Di-p53-1-6 and *h*DM2. In the case of CC-Di-p53-1-3, the grafted hotspots are at positions in the heptad that influence assembly, so these are more likely to dissociate in order to bind to *h*DM2. For CC-Di-p53-4 and CC-Di-p53-5, coiled coil dissociation on *h*DM2 binding may also occur; however, native MS shows that 1 : 1, 2 : 1 and 2 : 2 (peptide/protein) complexes are all feasible (Fig. S3). This is consistent with the AlphaFold models. CC-Di-p53-4-6 were less helical, less thermally stable or both, whilst the absence of stabilising interactions in the 2 : 2 complex and steric effects may favour a broader range of assembly states.

## Conclusions

In this study we have further probed the scope of parallel, homodimeric coiled coils as a scaffold in the design of PPI inhibitors. Our biophysical analyses conclude that the strategy can be adapted for the p53/*h*DM2 system to generate a set of α-helical ligands where substitution of the parent CC-Di at the *e* position of the heptad (close to the dimerization interface) is tolerated. Coiled-coil stability is closely linked to the extent of scaffold decoration and not only the positions on the heptad where grafting occurs. AlphaFold3 models of representative *h*DM2 complexes with CC-Di-p53-1 and CC-Di-p53-5 suggest grafting of p53 hot residues at *c* and *f* positions of the heptad may allow access to higher order oligomers to recruit an additional unit of protein, but that grafting at *e* positions of the heptad (CC-Di-p53-1) is not well tolerated, and the homodimer is likely to dissociate to bind in a 1 : 1 complex with the protein. Such a property could be harnessed, *i.e.* to switch between coiled coils and peptide/protein recognition in future studies. For the designs where residues were grafted only at *b*, *c* and *f* positions across heptads 2 and 3, *e.g.* CC-Di-p53-4-6, the assembly state of the coiled coils when bound to *h*DM2 and the complex stoichiometries are more diverse. This may indicate a requirement for high potency co-operative recognition in order to form higher order (*e.g.* 2 : 2) ternary complexes, but also a likely requirement for steric complementarity. Finally, as the scaffold was increasingly substituted with *h*DM2 binding residues, inhibitory potency of the peptides increased (*e.g.* CC-Di-p53-6 IC_50_ = 0.24 ± 0.03 µM). Selective recognition of *h*DM2 was also achieved over two other proteins (BCL-x_L_ and SPOP). To improve the therapeutic relevance of this technique in the future, surface-charge optimisation or the inclusion of a solubility tag may address the poor solubility of the designs in aqueous environments. Overall, these data provide valuable insight on the subtle interplay between coiled-coil assembly and recognition of target proteins by grafted residues to inform future design of parallel coiled-coil based PPI inhibitors.

## Author contributions

T. A. E., L. I., D. N. W., and A. J. W. conceived and designed the research programme and acquired funding. F. S., A. M. A.-J., D. G., and B. M. designed studies. F.S. performed biophysical analyses, A. M. A.-J. performed protein expression and biophysical analyses, D. G. prepared and purified peptides, and B. M. performed computational design. N. J. B. and A. C. L. performed mass spectrometry analysis. The manuscript was written and edited by F. S. and A. J. W. with contributions from all authors. All authors have given approval to the final version of the manuscript.

## Conflicts of interest

There are no conflicts to declare.

## Supplementary Material

CB-007-D6CB00063K-s001

## Data Availability

All relevant data are included in the supplementary information (SI). Supplementary information is available. See DOI: https://doi.org/10.1039/d6cb00063k.
