# Supplementary material for: De novo grafted coiled-coil peptides as p53/hDM2 inhibitors
Source: RSC Chem Biol. 2026 Jun 11. Online ahead of print. doi: 10.1039/d6cb00063k (PMC13256098; doi:10.1039/d6cb00063k)
Supplement: CB-OLF-D6CB00063K-s001 [file CB-OLF-D6CB00063K-s001.pdf]

Supplementary Information (SI) for RSC Chemical Biology.
This journal is © The Royal Society of Chemistry 2026

# Supporting Information for:

# *De Novo* Grafted Coiled-coil Peptides as p53/*h*DM2 Inhibitors

Freya Spain,[1] Diana Gimenez,[1] Amanda M. Acevedo-Jake,[1] Bram Mylemans,[2,3] Nikolas J. Brooks,[4] Boguslawa Korona,[5] Danny T. Huang,[6,7] Thomas A. Edwards,[8,9] Aneika C. Leney,[4] Laura Itzhaki,[5] Derek N. Woolfson,[2,3,10,11] Andrew J. Wilson*[1,9,12]

[1]School of Chemistry, University of Birmingham, Edgbaston, B15 2TT, UK

[2]School of Chemistry, University of Bristol, Cantock's Close, Bristol BS8 1TS, UK

[3]Novo Nordisk Foundation Center for Protein Design, Department of Biology and Department of Drug Design, University of Copenhagen, Copenhagen, Denmark

[4]School of Biosciences, University of Birmingham, Edgbaston, B15 2TT, UK

[5]Department of Pharmacology, University of Cambridge, Cambridge CB2 1PD, UK

[6]Cancer Research UK Scotland Institute, Garscube Estate, Switchback Road, Glasgow G61 1BD, UK

[7]School of Cancer Sciences, University of Glasgow, Garscube Estate, Switchback Road, Glasgow G61 1QH, UK

[8]College of Biomedical Sciences, Larkin University, 18301 N Miami Ave #1, Miami, Florida, 33169 USA

[9]Astbury Centre for Structural Molecular Biology, University of Leeds, Woodhouse Lane, Leeds LS2 9JT, UK

[10]School of Biochemistry, University of Bristol, Medical Sciences Building, University Walk, Bristol BS8 1TD, UK

[11]Max Planck-Bristol Centre for Minimal Biology, University of Bristol, Cantock's Close, Bristol BS8 1TS, UK

[12]School of Chemistry, University of Leeds, Woodhouse Lane, Leeds LS2 9JT, UK

## Table of Contents

Protein Overexpression and Purification ........ 8
Fluorescence Anisotropy Competition Assays ........ 13
Circular Dichroism ........ 13
Peptide Synthesis ........ 14
Analytical HPLC and MS of peptides ........ 15

## Supplementary Tables and Figures

### Table S1

| CC Position | Natural p53 | 3G03[1] | 3IUX[2] | 3JZS[3] | 3LNZ[4] | 4HFZ[5] | 5UMM |
|---|---|---|---|---|---|---|---|
| f | F | F (20) | F (21) | F (20) | F (20) | F (20) | F (20) |
| g | S | E (0) | L (6) | E (1) | A (/) | S (-2) | E (0) |
| a | D | / | / | / | / | / | / |
| b | L | Y (10) | Y (11) | W (7.5) | Y (9) | L (4) | Y (11) |
| c | W | W (23) | W (23) | W (23) | W (22) | W (25) | W (21) |
| d | K | / | / | / | / | / | / |
| e | K | A (/) | R (/) | S (/) | A (/) | K (/) | A (/) |
| f | L | L (9) | L (9) | L (8) | L (8) | L (8) | L (9) |
| g | P | T (1) | Q (4) | L (6) | S (1) | P (3) | M (4) |

PDBs were analysed with BAlaS[6] and at each position the residue and DDG value (kJ/mol) are shown in the table with positive values indicating the additional positive binding contribution compared to an Alanine at that position. The green highlighted cells correspond to the selected amino acid in the optimised peptide.

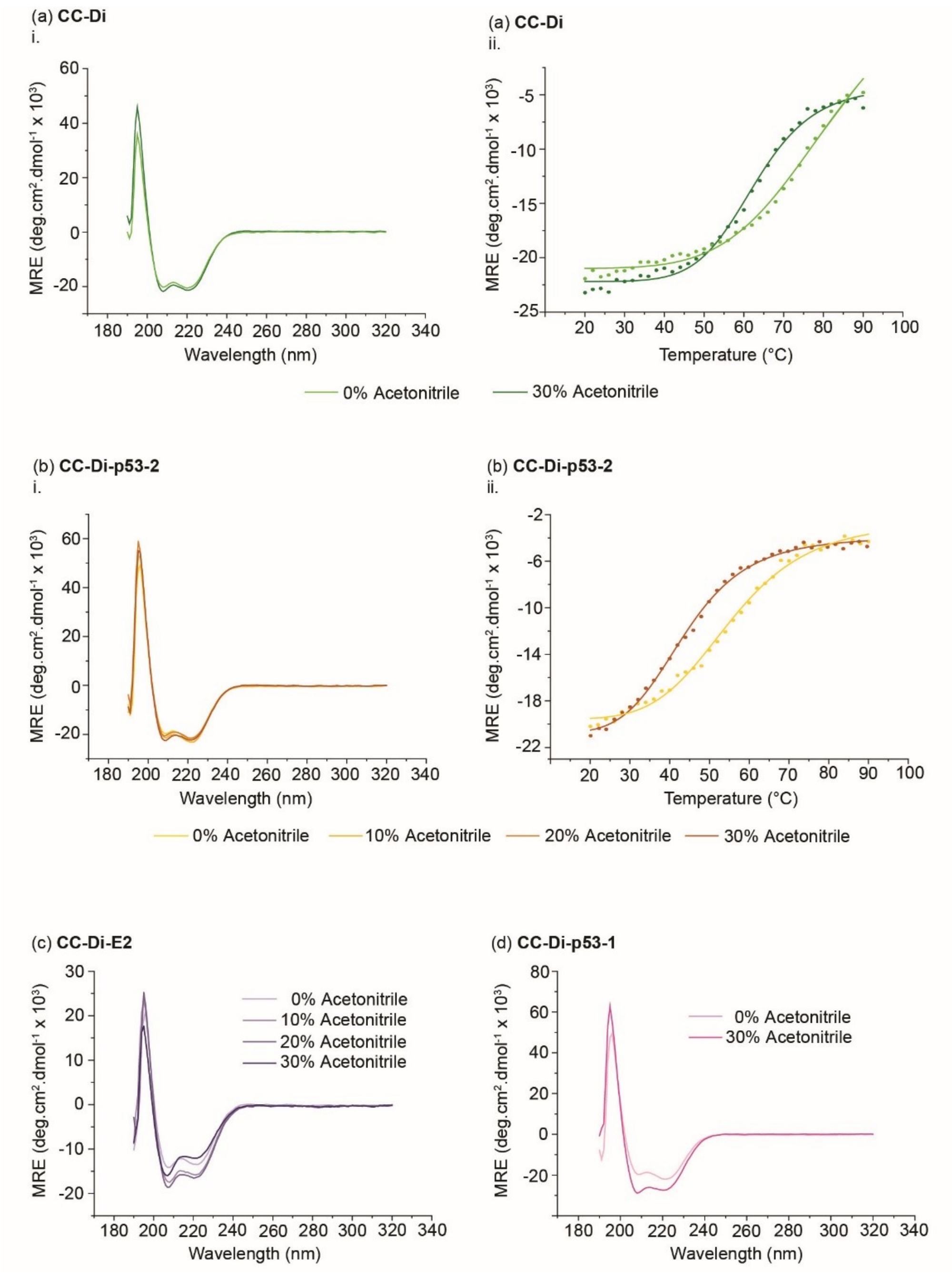


**Figure S1. CD analyses showing the effect of acetonitrile on coiled coil association.** (a) CC-Di parent scaffold (i) wavelength scans and (ii) thermal denaturation experiments without ($T_m$: 75 °C) and with 30% ($T_m$: 63 °C) acetonitrile, (b) representative peptide from the study, CC-Di-p53-2 (i) wavelength scans with varying concentrations of acetonitrile and (ii) thermal denaturation experiments without ($T_m$: 56 °C) and with 30% ($T_m$: 44 °C) acetonitrile. These data suggest that addition of acetonitrile to the buffer does effect coiled coil stability, decreasing the thermal midpoints of unfolding, however it has minimal effect on coiled coil association itself at 20 °C. Spectral scans of (c) a MCL-1 targeting coiled coil CC-Di-E2 and (d) a second representative *h*DM2 targeting coiled coil CC-Di-p53-1 show that despite the effect of increasing percentages of acetonitrile on the secondary structure of CC-Di-E2, the use of 30% acetonitrile to aid solubility had a negligible effect on the structure of the p53

coiled coil hybrids used in this work. Conditions: 20 µM peptide, 1mm cell, PBS pH 7.4 with varying percentages of acetonitrile, spectral scans performed at 25 °C.

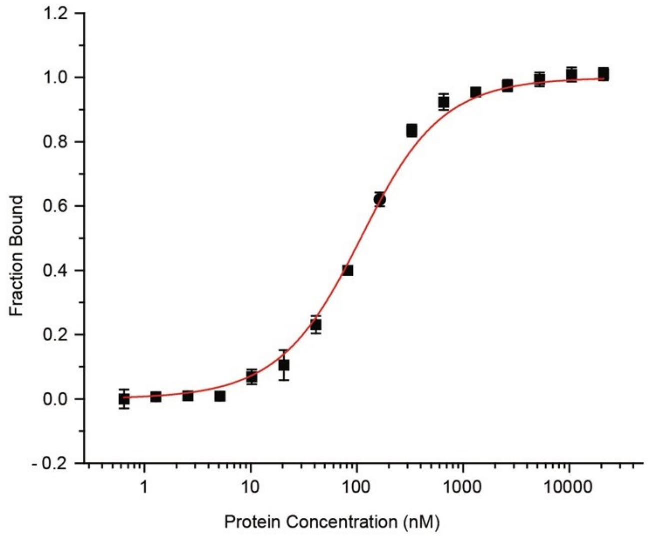


**Figure S2. Fluorescence anisotropy direct binding assay control of FAM-Ahx-p53-Opt/*h*DM2.** $K_d$ of p53-Opt was calculated to be 81 ± 5 nM. Conditions: 21 µM top concentration of *h*DM2, 54 nM FAM-Ahx-p53-T2, 25 °C, 20 mM Tris, 150 mM NaCl, pH 7.6.

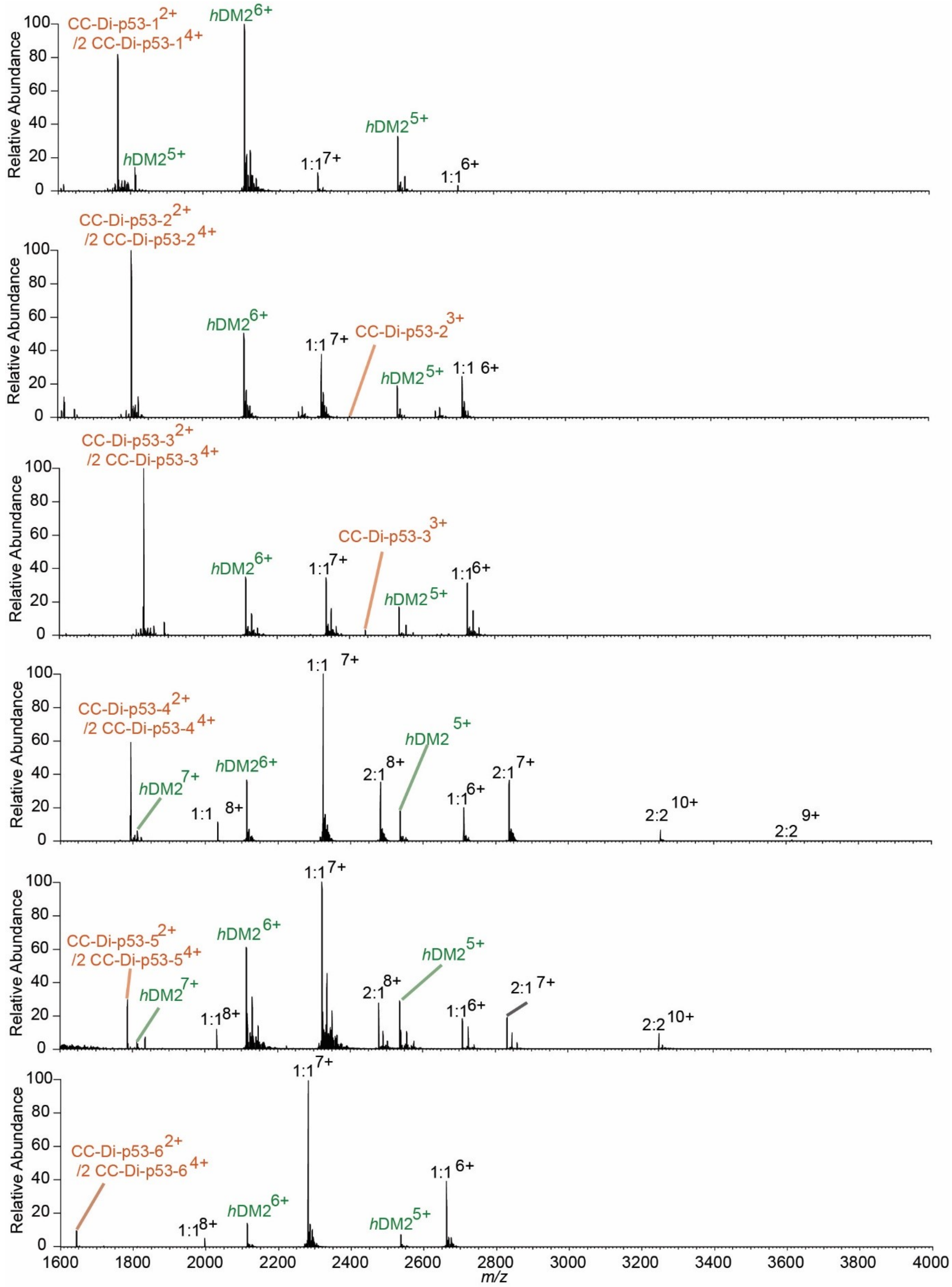


**Figure S3. Native mass spectrometry analyses of coiled-coil peptides with *h*DM2 protein;** orange indicates peaks corresponding to peptide alone, green indicates *h*DM2 protein peaks and black indicates peaks corresponding to complexes, with the stoichiometries displayed in panels samples analyzed at 5 μM of each component.

## Protein Overexpression and Purification

*h*DM2 was prepared and purified as described previously.[7] BCL-$x_L$ was prepared and purified as described previously.[8] The Plasmid for SPOP (pET28 vector, kanamycin resistance, residues 28 – 166, fusion protein $M_W$ = 18,098, fusion protein extinction coefficient = 24,200 $M^{-1}$ $cm^{-1}$, cleaved protein $M_W$ = 16,262 cleaved protein extinction coefficient = 22,710 $M^{-1}$ $cm^{-1}$) was transformed into the C41 *E. coli* strain, plated on LB-agar kanamycin selection plates, and single colonies used to make glycerol stocks. This stock was used to inoculate small overnight starter cultures (1:100 dilution into expression media, all media as LB broth containing 50 mg/mL kanamycin). Expression media was incubated at 37 °C until $OD_{600}$ reached 0.6; the temperature was then lowered to 18 °C and 800 μM IPTG added, and the protein overexpressed overnight. The next day, cells were pelleted (30 min, 5,000 rpm, Beckman Coulter JXN-26 floor centrifuge, fixed-angle JLA-8.100 rotor), and collected and frozen overnight at -80 °C to improve lysis. Cells were thawed and re-suspended in lysis buffer (25 mM Tris, 500 mM NaCl, pH 8.0; generally 25 mL buffer per 1 L of culture) containing protease inhibitor (1 tablet per 6-8 L cell pellet, cOmplete, Mini, EDTA-free protease inhibitor cocktail, Roche), lysozyme (5 mg per 6-8 L cell pellet, Roche) and DNAse (3 mg per 6-8 L cell pellet, Roche) and the mixture stirred for 20 min before sonication in a salt/ice bath (Q500 sonicator with a 12 mm probe, QSonica, cycles of 4 sec on 8 sec off, 60% amplitude, 6 min total). The lysate was clarified via centrifugation (45 min, 15,000 rpm, Beckman Coulter JXN-26 floor centrifuge, fixed-angle JA-25.50 rotor), passed through a 0.2 μm filter and applied to a 5 mL His-trap HP column (Merck) pre-equilibrated with wash 1 buffer (10 mM imidazole, 25 mM Tris, 500 mM NaCl, pH 8.0). The column was then washed with wash 2 buffer (10 column volumes, 20 mM imidazole, 25 mM Tris, 500 mM NaCl, pH 8.0), wash 3 buffer (10 column volumes, 40 mM imidazole, 25 mM Tris, 500 mM NaCl, pH 8.0) and wash 4 buffer (10 column volumes, 100 mM imidazole, 25 mM Tris, 500 mM NaCl, pH 8.0) before elution (10 column volumes elution buffer, 400 mM imidazole, 25 mM Tris, 500 mM NaCl, pH 8.0). An SDS-PAGE gel (4-20% gradient gel, Bio-Rad Laboratories) confirmed the fractions containing the purified fusion protein, which were then pooled for overnight dialysis (4 L total volume, 20 mM Tris, 150 mM NaCl, pH 8.0, MWCO 10,000 Da dialysis tubing, Thermo Scientific) and cleavage of the N-terminal His-TEV affinity tag via His-TEV protease (1:20 ratio of protease:fusion protein). The next day the contents of the dialysis tubing were passed through a 0.2 μm filter before application to a 5 mL His-trap HP column. The flow through, containing the purified and TEV-cleaved protein, was collected, while uncleaved fusion protein, N-terminal His-TEV affinity tag and His-TEV protease remained bound to the nickel column. After confirmation via SDS-PAGE, the cleaved and purified protein was concentrated (10,000 MWCO Thermo Scientific) for size exclusion chromatography (HiLoad™ 26/60 Superdex 75 column, GE healthcare, Akta Prime Plus, 2 mL / min flow rate, 20 mM Tris, 250 mM NaCl, 0.5 mM DTT, 2.5% glycerol, pH 8.0), the fractions containing the purified protein collected, and the protein concentrated (generally 150 – 250 μM) for storage in at -80 °C. Before use in experiments, concentrations were calculated using the absorbance at A280 on a NanoDrop One (ThermoScientific).

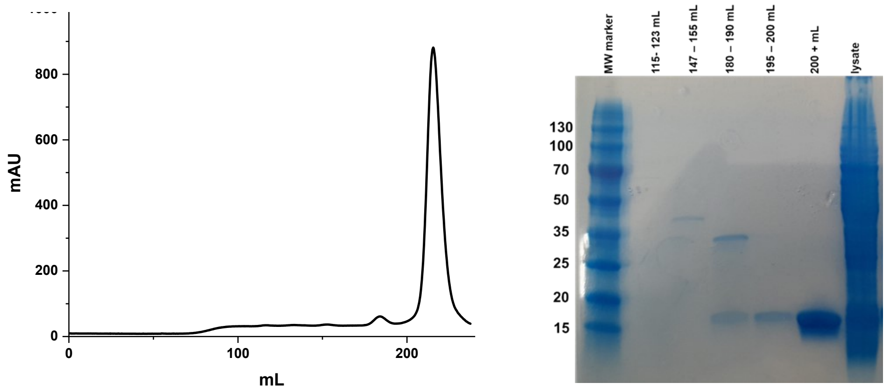


Size exclusion chromatogram of SPOP after affinity tag cleavage and the reverse nickel column (left); fractions of pure protein were collected from 200 – 220 mL. SDS-PAGE gel of SPOP after size exclusion chromatography (right).

## Direct Fluorescence Anisotropy Titration Assays

Direct binding assays were performed in 20 mM Tris, 150 mM NaCl, pH 7.4 in roundlow volume 384 well plates (Corning). Fluorescent peptide concentrations were fixed at 54.5 nM. Protein was titrated from the highest concentration with successive two-fold serial dilutions over 16 points, then fluorescent peptide (or for control wells, buffer) added. All experiments were performed in triplicate (for both the test wells with fluorescent tracer peptide and control wells lacking the fluorescent tracer), and plates were read after 1 h incubation at room temperature. Data was collected with a CALRIOstar plate reader (BMG Labtech), and excitation at 482 nm (bandwidth 16 nm) and emission at 530 nm (bandwidth 40 nm).

Perpendicular and parallel intensities of the control wells were averaged and deducted from each corresponding samples to give the corrected values, *P* and *S*. The total sample intensities (I) and anisotropies (r) were calculated (equations 1 and 2):

$$I = (2PG) + S\#() \quad 1$$

$$r = (S - PG)\#() \quad 2$$

Fluorescence anisotropy values were plotted against protein concentration and the data fit to a sigmoidal logistic model to obtain $r_{min}$ and $r_{max}$, then Ligand bound calculated and the $L_b$ was further determined (equation 3).

$$L_b = \frac{r - r_{min}}{\lambda(r_{max} - r) + r - r_{min}}\#() \quad 3$$

The $K_d$ was then calculated (equation 4):

$$y = \frac{(k + x + [FL]) - \sqrt{(k + x + [FL]^2 - 4 \times x \times [FL])}}{2}\#() \quad 4$$

I = total intensity; P = corrected perpendicular intensity; S = corrected parallel intensity; G = instrument factor which was set to 1.0 for all assays; r = anisotropy; $L_b$ = ligand bound fraction; λ = 1, [FL] = fluorescent ligand concentration; k = $K_d$; y = $L_b$ × [FL] and x = added protein concentration.

## Fluorescence Anisotropy Competition Assays

Competition assays were performed in 20 mM Tris, 150 mM NaCl, pH 7.4 or pH 7.6 (indicated where appropriate) in round bottom low volume 384 well plates (Corning). Peptide stocks were prepared in the assay buffer with minimal DMSO and diluted across the plate (0.5 serial dilution). *h*DM2 concentrations were fixed at 110 nM and the tracer FAM-Ahx-p53-Opt fixed at 54.5 nM. All experiments were performed in triplicate (for both the test wells with fluorescent tracer peptide and control wells lacking the fluorescent tracer), and plates were read after 1 h incubation at room temperature. Data was collected with a CALRIOstar plate reader (BMG Labtech), and excitation at 482 nm (bandwidth 16 nm) and emission at 530 nm (bandwidth 40 nm).

Perpendicular (P) and parallel (S) intensities together with total sample intensities (I) and anisotropies (r) were calculated as before, then fluorescence anisotropy values were normalized from 0 to 1, and plotted against peptide competitor concentrations and fit to a logistic function (5) to determine the half-maximal inhibitory concentration ($IC_{50}$) of the peptide competitor

$$y = r_{max} + \frac{r_{min} - r_{max}}{1 + (x/x_0)^p} \#() \quad 5$$

$x_0$ is the midpoint or $IC_{50}$ and $p$ is the power

## Circular Dichroism

Peptide stocks were prepared in 30% acetonitrile, PBS pH 7.4 with concentrations calculated from UV absorption at 280 nm ($\varepsilon$ = 6990 $mol^{-1}cm^{-1}$ for each peptide) using a Varian Cary 50 UV-Vis Spectrophotometer. The stocks were diluted down in the same buffer (unless otherwise stated) to a final concentration of 20 µM. Wavelength spectral scans were collected at 25 °C (experiment ran in duplicate, each averaged over 3 scans, 320 - 190 nm), scanned at 50 nm/min with a 1 nm data pitch and 4 s D.I.T. Thermal denaturation experiments were conducted from 20 – 90 °C, ramping at 1 °C per minute with a temperature halt at each data point. The CD signal at 222 nm was recorded across the temperature range at 2 °C intervals (2 nm bandwidth, 4 s D.I.T, 30 s response time). The buffer baseline was recorded using the same parameters as the samples and was subtracted from each measurement. $T_m$ was calculated from the mid-point of unfolding transitions.

Data were collected on a Jasco J-1500 CD Spectrometer using 1 mm pathlength quartz cuvettes. Raw ellipticity data were converted to mean residue ellipticity (MRE) using the following equation:

$$MRE = (([\theta]\lambda - [\theta]0)\, x\, Mw) / (n\, x\, l\, x\, c)$$

Where $[\theta]_\lambda$ is the observed ellipticity at a wavelength λ in mdeg, $[\theta]_0$ is the ellipticity observed for the matched buffer, $M_w$ = molecular weight (g $mol^{-1}$), *n* = number of amide bonds, *c* = sample concentration (mg $mL^{-1}$); *l* = pathlength of the cuvette in cm.

## Native Mass Spectrometry

Lyophilised peptides were reconstituted in either 100 mM ammonium acetate (pH 6.8) or a minimum volume of HPLC grade DMSO, before dilution to a stock concentration of 100 µM in 100 mM ammonium acetate (pH 6.8). *h*DM2 was exchanged into 100 mM ammonium acetate (pH 6.8) using a 10 kDa MWCO Amicon Ultra 0.5 mL centrifugal concentrator. Peptide stock solutions and *h*DM2 were stored at -20$^{o}$C prior to use.

*h*DM2 – peptide mixtures were each analysed at a 1:1 molar ratio (5 µM *h*DM2: 5 µM peptide). Native mass spectrometry analysis was performed using a Q Exactive HF mass spectrometer (Thermo Fisher Scientific) equipped with a nanoelectrospray ionisation source. Samples were introduced into the mass spectrometer using gold-coated borosilicate capillaries, prepared in-house. Positive ionisation mode was used throughout, with the capillary voltage set to 1.1 kV. The source temperature was set to 250$^{o}$C, in-source fragmentation at 0 V, and S-lens RF level 100. Mass spectra were acquired with an absolute automatic gain control target of $5e^{6}$, maximum ion injection time of 100 ms, and 10 micro-scans were acquired per scan cycle. Ions were detected in the Orbitrap at a resolution of 15,000 at *m/z* 200. The mass range was set to 700 – 6000 *m/z*. All data was analysed using Xcalibur (*v*4.2). The observed molecular weights for all detected *h*DM2-peptide complexes were calculated from a minimum of two charge states.

**Table S2. Average theoretical and observed molecular weights of peptides and peptide-*h*DM2 complexes detected by native MS**. The error on the masses calculated across charge states in all cases was ≤ 0.3 Da. The mono-isotopic and average isotopic masses are reported for peptide and protein/protein-peptide complexes, respectively.

| Peptides/*h*DM2 complexes | Theoretical mass (Da) | Observed mass (Da) |
|---|---|---|
| CC-Di-p53-1 | 3526.92 | 3526.93 |
| CC-Di-p53-2 | 3603.89 | 3603.87 |
| CC-Di-p53-3 | 3661.89 | 3661.92 |
| CC-Di-p53-4 | 3584.95 | 3584.97 |
| CC-Di-p53-5 | 3568.99 | 3569.02 |
| CC-Di-p53-6 | 3284.84 | 3284.87 |
| 2 CC-Di-p53-1 | 7053.84 | 7053.79 |
| 2 CC-Di-p53-2 | 7207.77 | 7207.69 |
| 2 CC-Di-p53-3 | 7323.79 | 7323.79 |
| 2 CC-Di-p53-4 | 7169.90 | 7169.94 |
| 2 CC-Di-p53-5 | 7137.98 | 7138.01 |

| | | |
|---|---|---|
| 2 CC-Di-p53-6 | 6569.69 | 6569.73 |
| *h*DM2 | 12680.5 | 12680.5 |
| *h*DM2:CC-Di-p53-1 | 16206.1 | 16206.3 |
| *h*DM2:CC-Di-p53-2 | 16284.4 | 16284.8 |
| *h*DM2:CC-Di-p53-3 | 16342.4 | 16342.3 |
| *h*DM2:CC-Di-p53-4 | 16265.4 | 16265.4 |
| *h*DM2: 2 CC-Di-p53-4 | 19850.4 | 19850.6 |
| 2 *h*DM2: 2 CC-Di-p53-4 | 32530.8 | 32530.6 |
| *h*DM2:CC-Di-p53-5 | 16249.5 | 16249.5 |
| 2 *h*DM2:CC-Di-p53-5 | 19818.4 | 19818.5 |
| 2 *h*DM2: 2 CC-Di-p53-5 | 32498.9 | 32498.5 |
| *h*DM2:CC-Di-p53-6 | 15965.3 | 15965.3 |

## Peptide Synthesis

### General materials and methods

All Fmoc-protected amino acids and coupling reagents were purchased from Fluorochem. DMF used for peptide synthesis was ACS grade from VWR and solvents used for purification were HPLC quality and provided by Sigma Aldrich or Fisher.

Preparative purifications have been performed using RP-HPLC using an Ascentis C18 (250mm x 21.2 mm, 5µm, Supelco) column at 15 ml/min flow rate. Eluents employed were A: 0.1% FA in $H_2O$ and B: 0.1% FA acid in acetonitrile using a gradient of 5-30 or 10-30% B over 45 minutes. Pooled fractions were analyzed using LC-(ESI+)MS (Eluents were A: 0.1% formic acid in H2O and B: 0.1% formic acid in acetonitrile), and fractions corresponding to single chromatographic peaks combined together and freeze dried.

To confirm the final purity of each peptide, analytical RP-HPLC analyses were performed on a Shimadzu LCMS-2050 system using an Ascentis C18 (250mm x 4.6 mm, 5µm, Supelco) column at 1.5 ml/min flow rate and diode array detection at $\lambda$ = 214 nm. In addition, high-resolution mass spectrometry analyses, (HR-(ESI+)LC/MS), used to confirm the identity of each peptide, were performed on Bruker maXis II™ ESI–QToF mass spectrometer.

### Solid-phase peptide synthesis

All peptides were automatically synthesized in a Chorus9 SPPS synthesizer using Rink Amide resin (238 mg, loading 0.42 mol $g^{-1}$, 0.1 mmol scale) in the presence of 5 eq. amino acid excess using DIC and Oxyma (5 mol eq. each) as coupling reagents. For each amino-acid, double deprotections (2 x 2 min) followed by single or double

couplings (4 min) were performed at 75 °C. Final deprotection and acetylation of the sequences were accomplished by double treatment with 20/0.5% piperidine/formic acid *v/v* in DMF for 15 minutes followed by treatment of the peptide-resin with 50 mol equivalents of acetic anhydride and DIPEA in DMF (2 x 1mL x 30 minutes).

For FAM-Ahx- labelled peptides, once the synthesis was completed, 5 mol eq. of Fmoc-Aminohexanoic acid, 5 eq. of OXYMA and 5 eq. of DIC were added to each resin and the mixtures left to react on the rotary shaker for 1 h. Once completed, the solutions were drained and the resins thoroughly washed with DMF (4 x 5 mL x 2min). The Fmoc group was then removed using 2 mL of a 20% piperidine solution in DMF (2 x 3 mL x 15 min) and extensive DMF washes repeated (4 x 5 mL x 2 min). Then, 3 mol eq. of (5/6)-FAM, DIC and OXYMA were added, and the mixtures were left to react in the rotary shaker (protected from any source of light) overnight. Upon completion, the mixtures were drained and the resins washed sequentially with DMF (2 x 5 mL x 2 min), 20% piperidine in DMF (2 x 3 mL x 20 min) and $CH_2Cl_2$ (4 x 5 mL x 2 min), before being finally left to dry under reduced pressure.

Peptides were cleaved using a TFA:TIPS:$H_2O$:DODT 95:2.5:2.5:2.5 solution for 3 hours at room temperature (2 mL per 100 mg of peptide-resin). The cleavage cocktails were filtered over $Et_2O$ (30 mL) and the final crude materials left to precipitate overnight at -20 °C. The precipitated peptides were recovered by centrifugation (6000 rpm; 4 min) and washed with pre-chilled $Et_2O$ (-20 °C; 2 x 15 mL). Finally, crude materials were dissolved in a mixture of $H_2O$:MeCN 75:25% v/v and lyophilized.

## Analytical HPLC and MS of peptides

**Ac-CC-Di-p53-1**

**Sequence:**

Ac- GEIAALFQEIWALLKENAALKQEIAALKQGYG -$CONH_2$

HPLC (λ = 214 nm) tR = 13.898 min. HR-QToF (ESI) m/z calc. for $\mathbf{C_{163}H_{259}N_{41}O_{46}}$: $[M+2H]^{2+}/[2M+4H]^{4+}$ Calc: 1764.4667, Found: 1764.4564; $[M+3H]^{3+}/[2M+6H]^{6+}$: Calc: 1176.6469, Found: 1176.6453. $[2M+5H]^{5+}$: Calc: 1411.7748, Found:1411.7587; $[M+4H]^{4+}/[2M+8H]^{8+}$: Calc: 882.7370, Found: 882.7282.

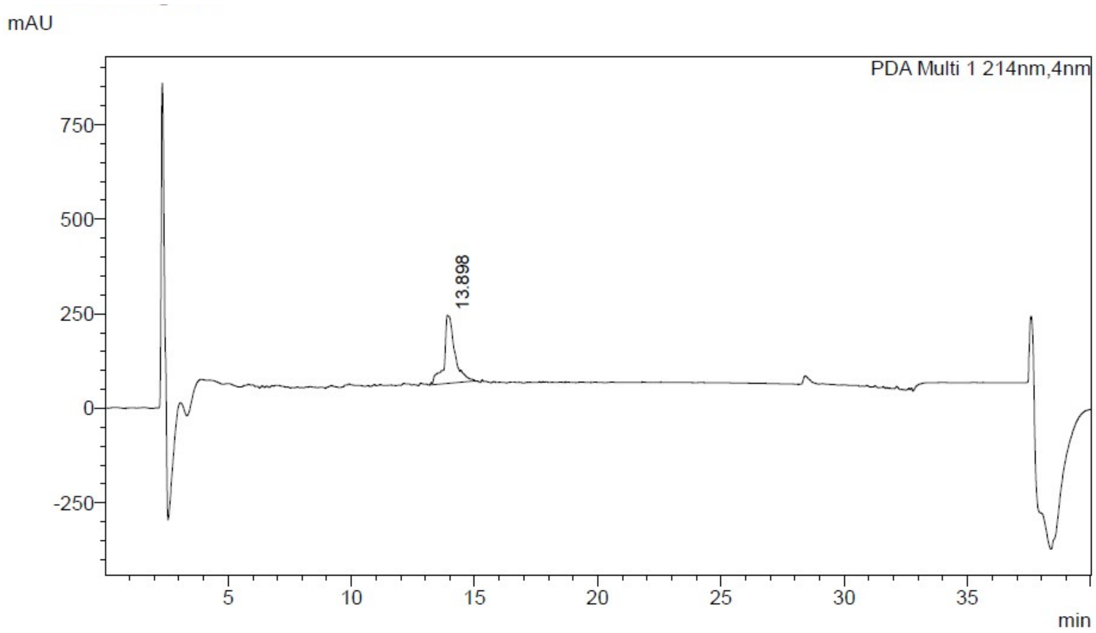


**Analytical HPLC trace at λ= 214 nm of purified Ac-CC-Di-p53-1.**

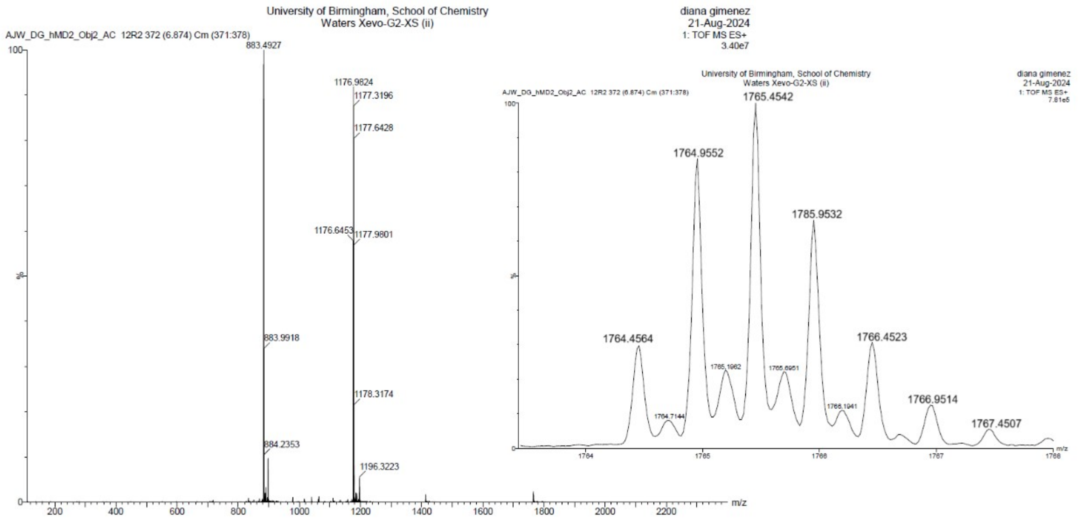


**HR-QToF(ESI+)MS analysis of purified Ac-CC-Di-p53-1.**

## FAM-Ahx-CC-Di-p53-1

### Sequence:

FAM-Ahx-GEIAALFQEIWALLKENAALKQEIAALKQGYG -$CONH_2$

HPLC (λ = 214 nm) tR = 15.065 min. HR-QToF (ESI) m/z calc. for $\mathbf{C_{188}H_{278}N_{42}O_{52}}$: $[M+3H]^{3+}$: Calc: 1319.6873, Found: 1319.6873; $[M+4H]^{4+}$: Calc: 990.0173, Found: 990.0134; $[M+5H]^{5+}$: Calc: 792.2153, Found:792.2103.

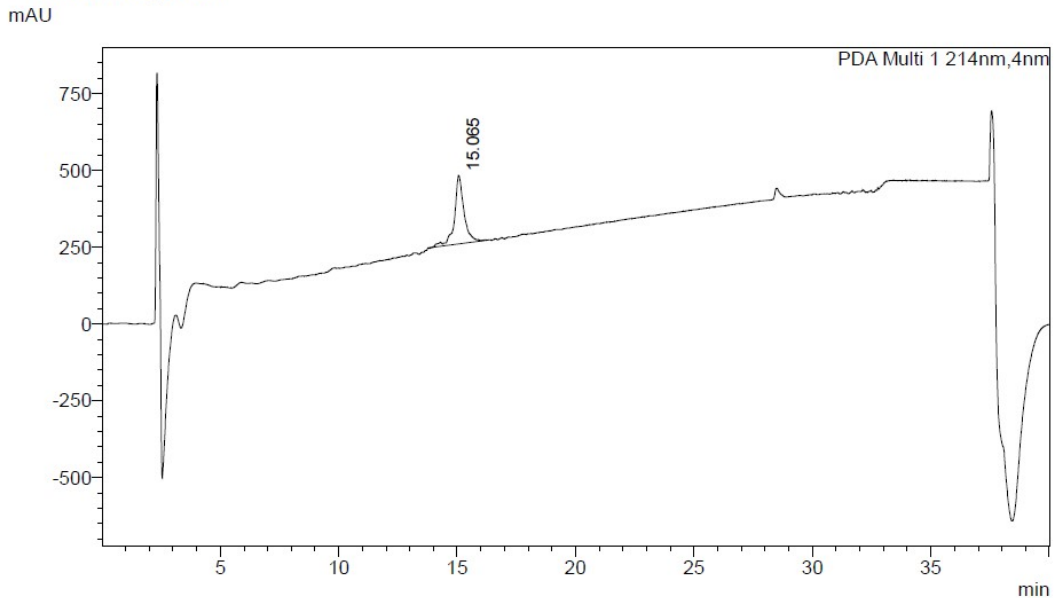


**Analytical HPLC trace at λ= 214 nm of purified FAM-Ahx-CC-Di-p53-1.**

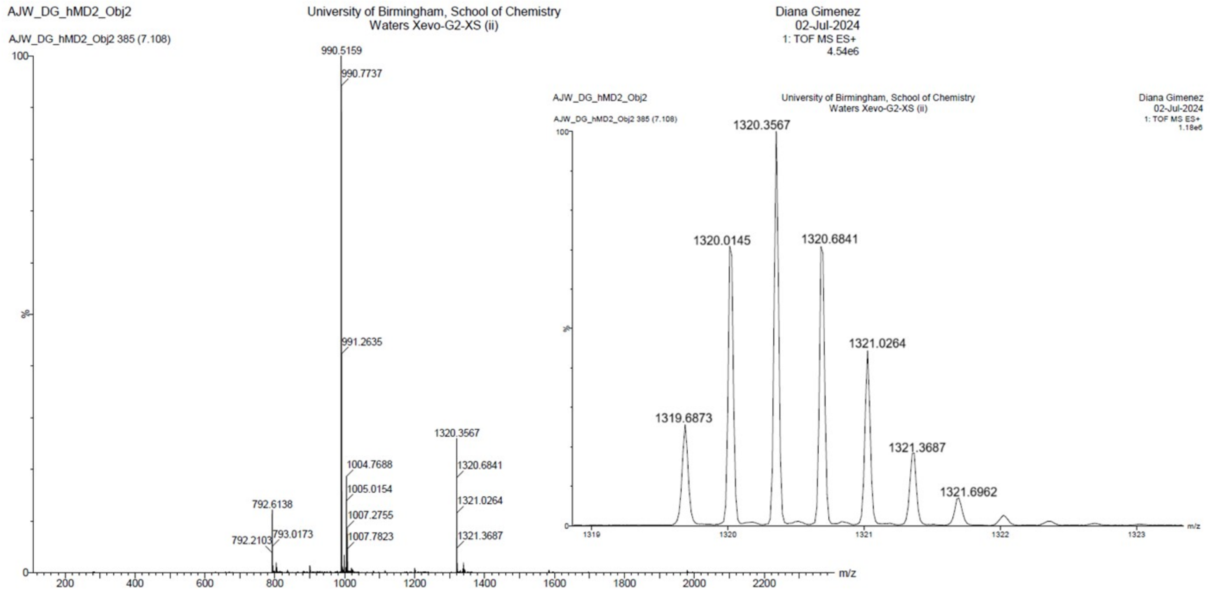


**HR-QToF(ESI+)MS analysis of purified FAM-Ahx-CC-Di-p53-1.**

**Ac-CC-Di-p53-2**

**Sequence:**

Ac- GEIAALFQEIWFLLEENAALKQEIAALKQGYG -$CONH_2$

HPLC (λ = 214 nm) tR = 4.710 min. HR-QToF (ESI) m/z calc. for $\mathbf{C_{168}H_{258}N_{40}O_{48}}$: $[M+2H]^{2+}/[2M+4H]^{4+}$ Calc: 1802.9561, Found: 1802.9370; $[M+3H]^{3+}/[2M+6H]^{6+}$: Calc: 1202.3065, Found: 1202.2926; $[2M+5H]^{5+}$: Calc: 1442.5664, Found:1442.5437; $[M+4H]^{4+}/[2M+8H]^{8+}$: Calc: 901.9817, Found: 901.9802.

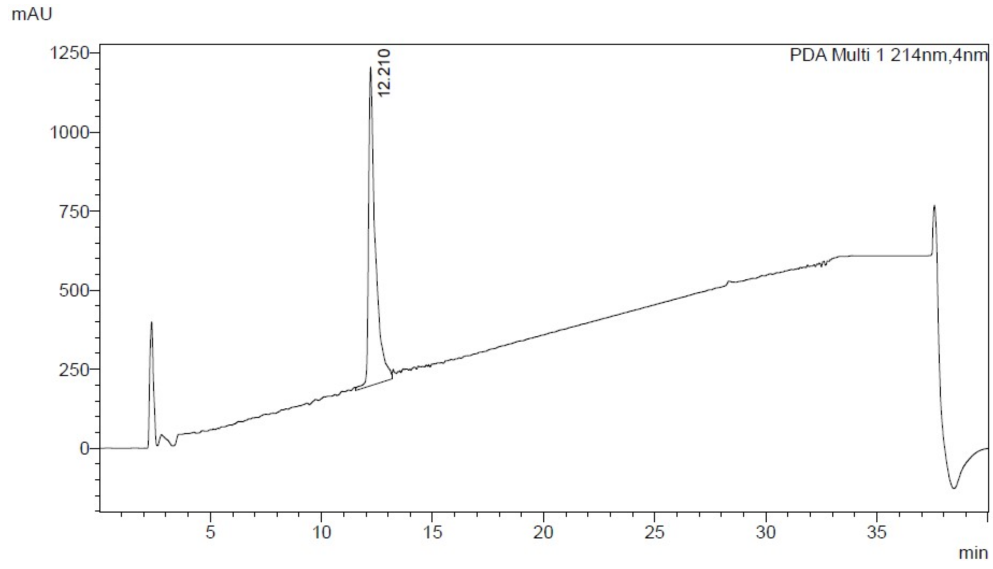


**Analytical HPLC trace at λ= 214 nm of purified Ac-CC-Di-p53-2.**

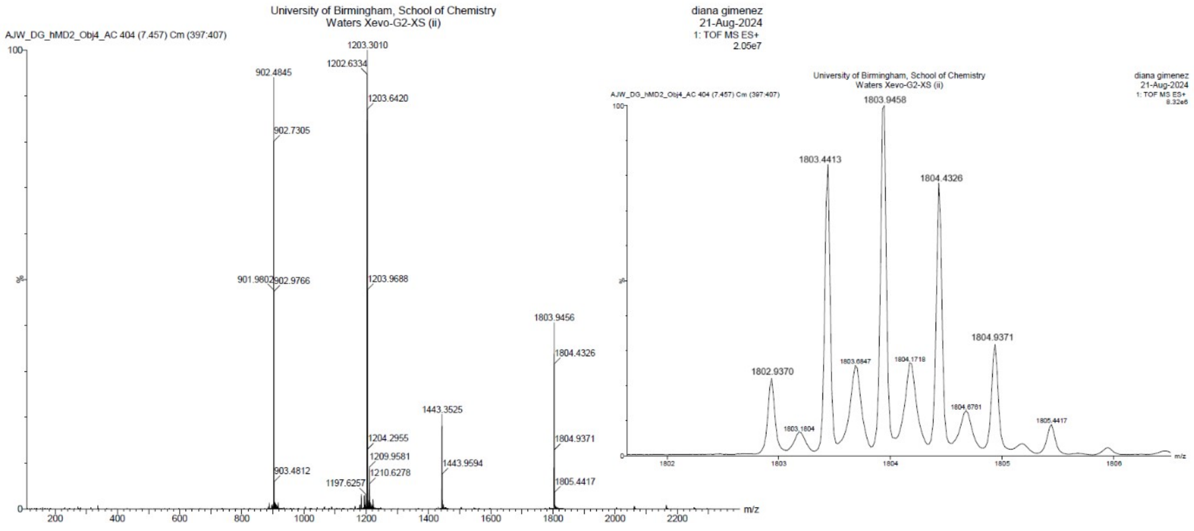


**HR-QToF(ESI+)MS analysis of purified Ac-CC-Di-p53-2.**

**FAM-Ahx- Ac-CC-Di-p53-2**

**Sequence:**

FAM-Ahx- GEIAALFQEIWFLLEENAALKQEIAALKQGYG -CONH$_2$

HPLC (λ = 214 nm) tR = 16.334 min. HR-QToF (ESI) m/z calc. for $\mathbf{C_{193}H_{277}N_{41}O_{54}}$: $[M+2H]^{2+}/[2M+4H]^{4+}$ Calc: 2017.5168, Found: 2017.5205; $[M+3H]^{3+}/[2M+6H]^{6+}$: Calc: 1345.3469, Found: 1345.3521; $[2M+5H]^{5+}$: Calc: 1614.2149, Found: 1614.2118; $[M+4H]^{4+}/[2M+8H]^{8+}$: Calc: 1009.2620, Found: 1009.2648.

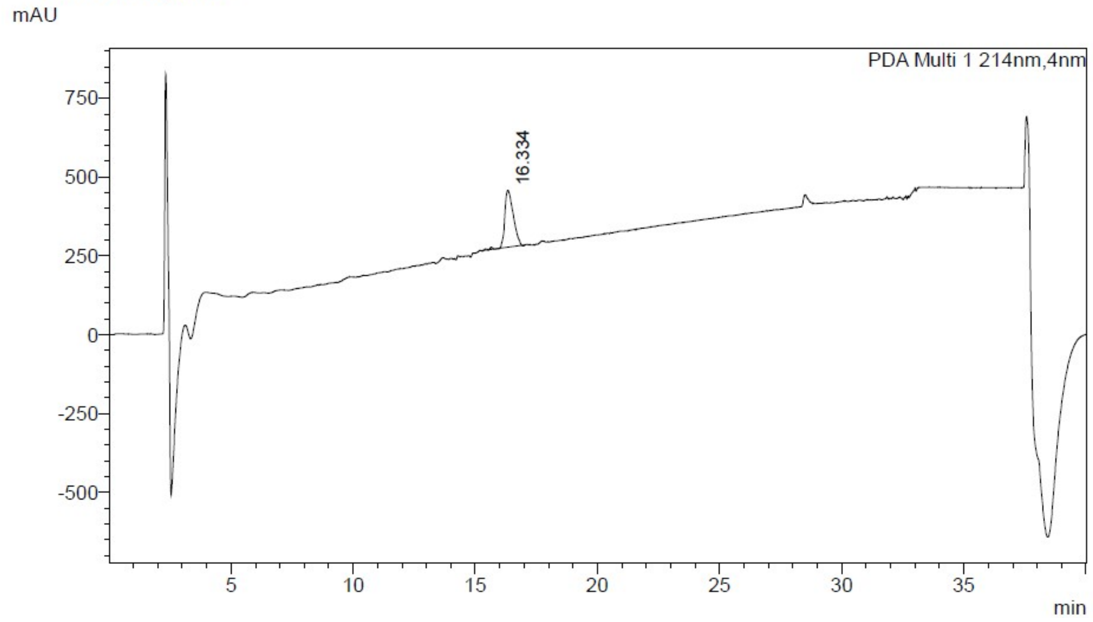


**Analytical HPLC trace at λ= 214 nm of purified FAM-Ahx-CC-Di-p53-2.**

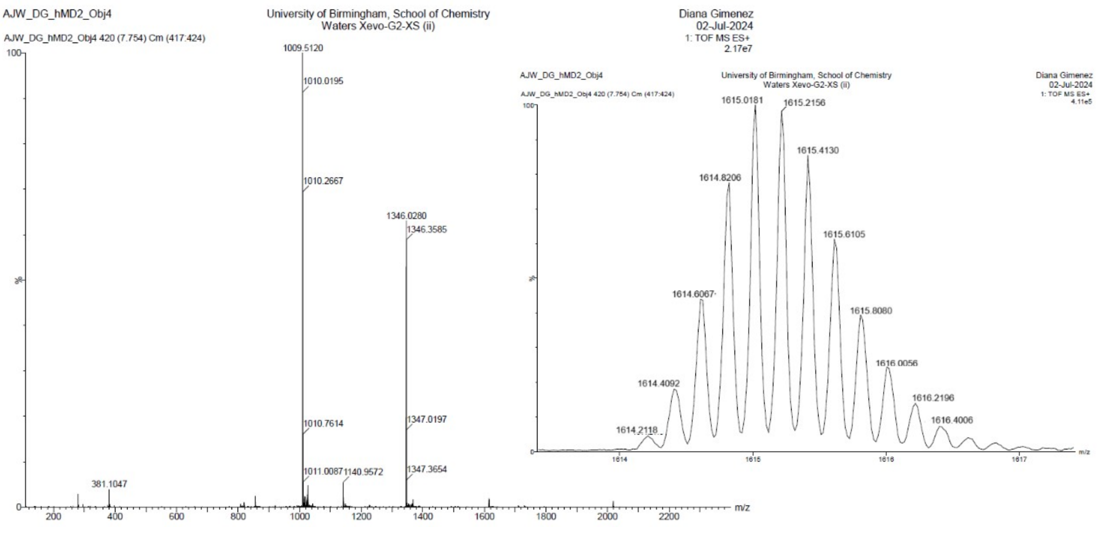


**HR-QToF(ESI+)MS analysis of purified FAM-Ahx-CC-Di-p53-2.**

**Ac-CC-Di-p53-3**

**Sequence:**

Ac- GEIEALFQEIWFLLEENAALKQEIAALKQGYG -$CONH_2$

HPLC (λ = 214 nm) tR = 14.661 min. HR-QToF (ESI) m/z calc. for $\mathbf{C_{170}H_{260}N_{40}O_{50}}$: $[M+2H]^{2+}/[2M+4H]^{4+}$: Calc: 1831.9589, Found: 1831.9515; $[2M+5H]^{5+}$: Calc: 1465.7686, Found: 1465.7641; $[M+3H]^{3+}/ [2M+6H]^{6+}$: Calc: 1221.6417, Found: 1221.6381; $[M+4H]^{4+}/ [2M+8H]^{8+}$: Calc: 916.4831, Found: 916.4889.

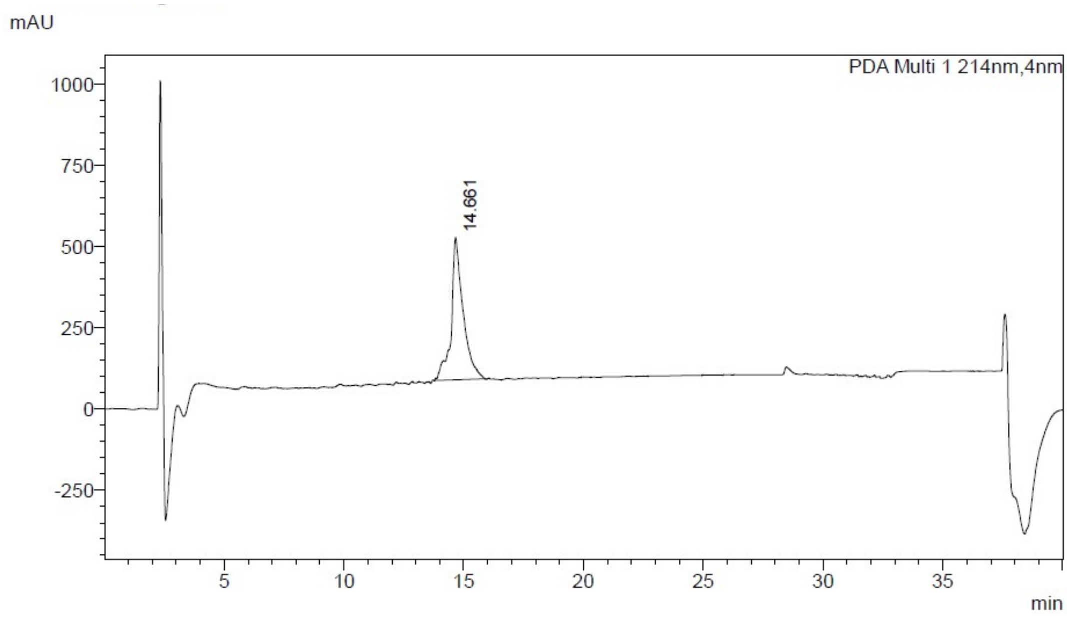


**Analytical HPLC trace at λ= 214 nm of purified Ac-CC-Di-p53-3.**

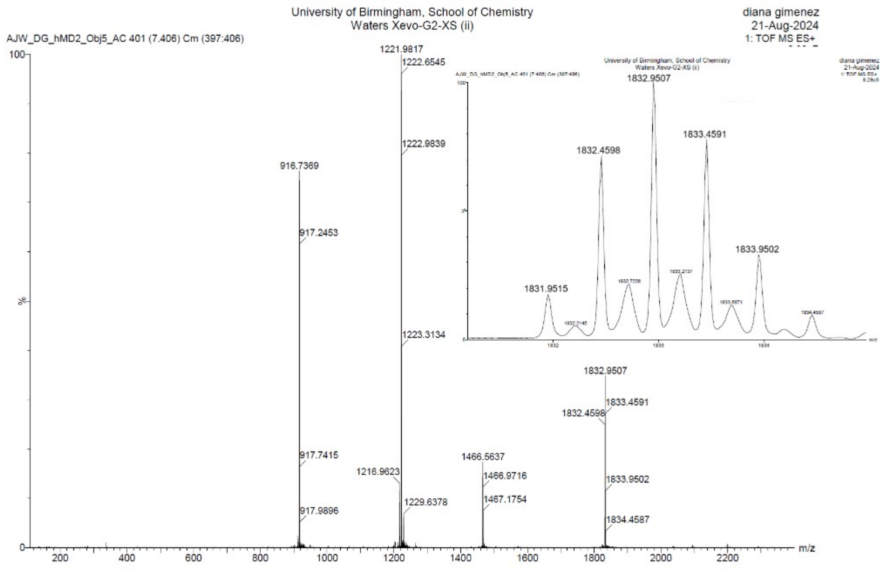


**HR-QToF(ESI+)MS analysis of purified Ac-CC-Di-p53-3.**

**FAM-Ahx-CC-Di-p53-3**

**Sequence:**

FAM-Ahx-GEIEALFQEIWFLLEENAALKQEIAALKQGYG-CONH$_2$

HPLC (λ = 214 nm) tR = 16.226 min. HR-QToF (ESI) m/z calc. for **$C_{195}H_{279}N_{41}O_{56}$**: $[M+3H]^{3+}$: Calc: 1364.6821, Found: 1364.6758; $[M+3Na]^{3+}$: Calc: 1386.6640, Found: 1386.7047; $[M+4H]^{4+}$: Calc: 1023.7634, Found: 1023.7694; $[M+3Na+H]^{4+}$: Calc: 1041.0057, Found: 1041.0046.

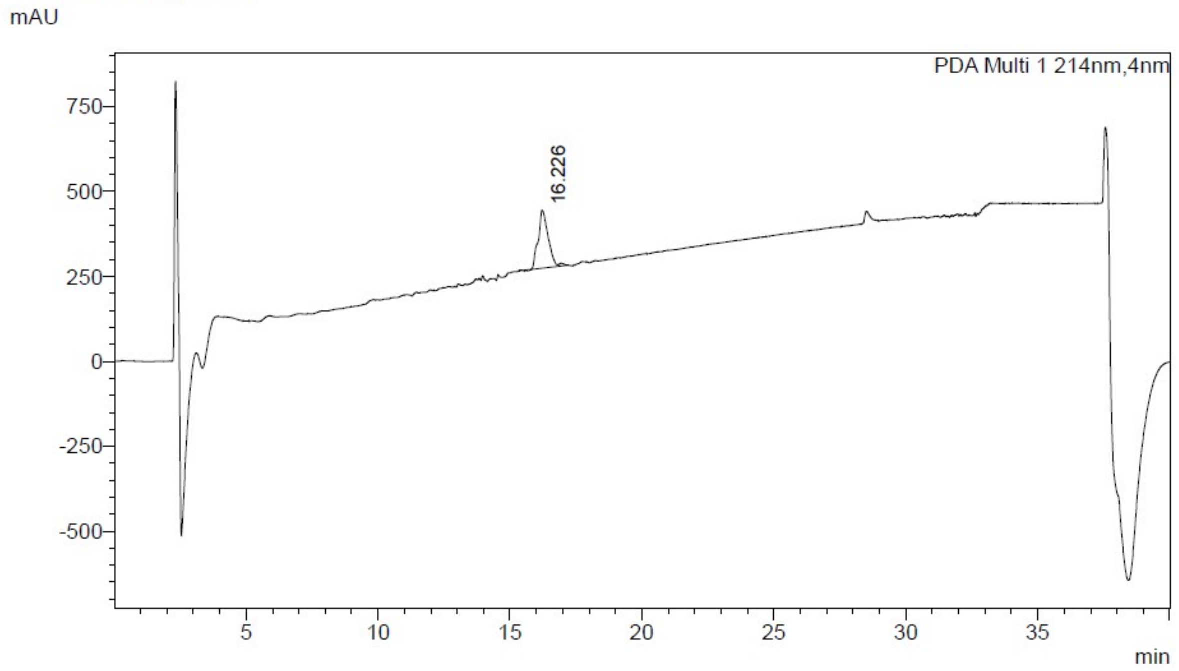


**Analytical HPLC trace at λ= 214 nm of purified FAM-Ahx-CC-Di-p53-3.**

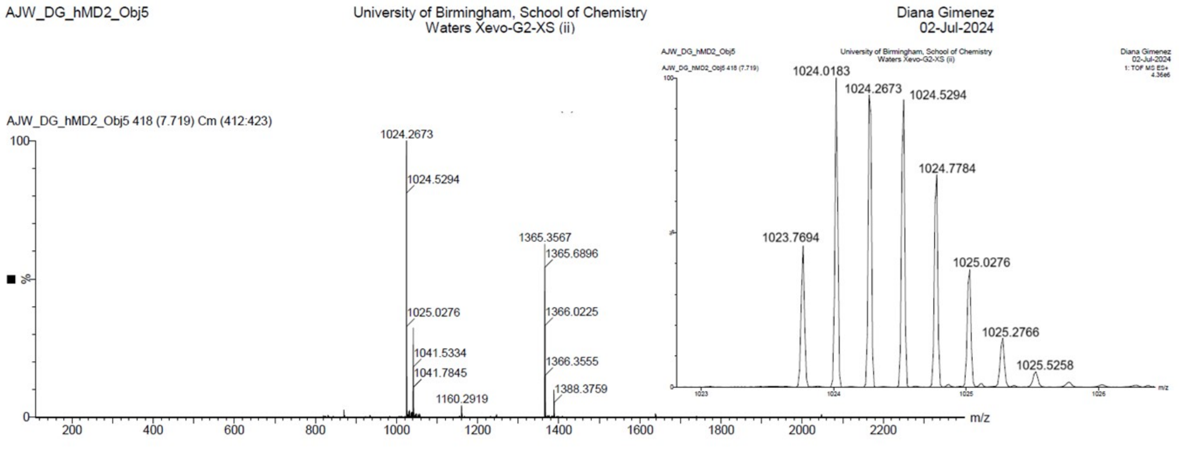


**HR-QToF(ESI+)MS analysis of purified FAM-Ahx-CC-Di-p53-3.**

**Ac-CC-Di-p53-4**

**Sequence:**

Ac- GEIAALKQEIAALKFENAWLKLEIEALKQGYG -$CONH_2$

HPLC (λ = 214 nm) tR = 11.755 min. HR-QToF (ESI) m/z calc. for $\mathbf{C_{166}H_{265}N_{41}O_{47}}$: $[M+2H]^{2+}$: Calc: 1793.4876, Found: 1793.4855; $[M+3H]^{3+}$: Calc: 1195.9942, Found: 1195.9908; $[M+4H]^{4+}$: Calc: 897.2474, Found: 897.2480.

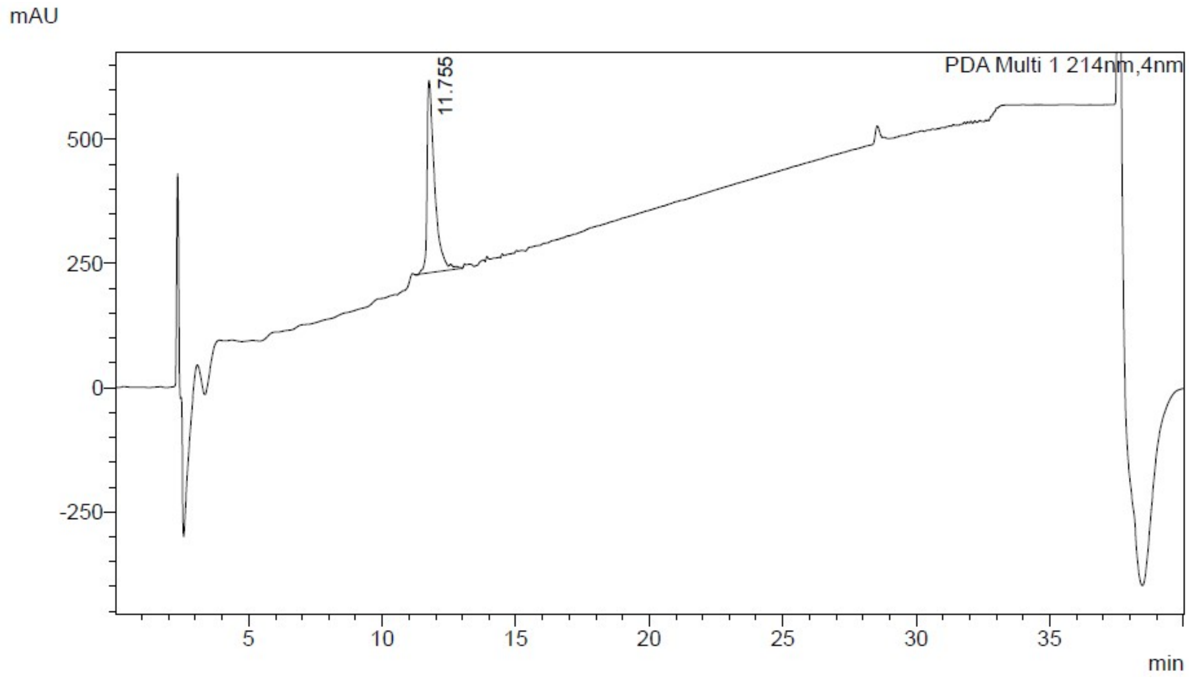


**Analytical HPLC trace at λ= 214 nm of purified Ac-CC-Di-p53-4**.

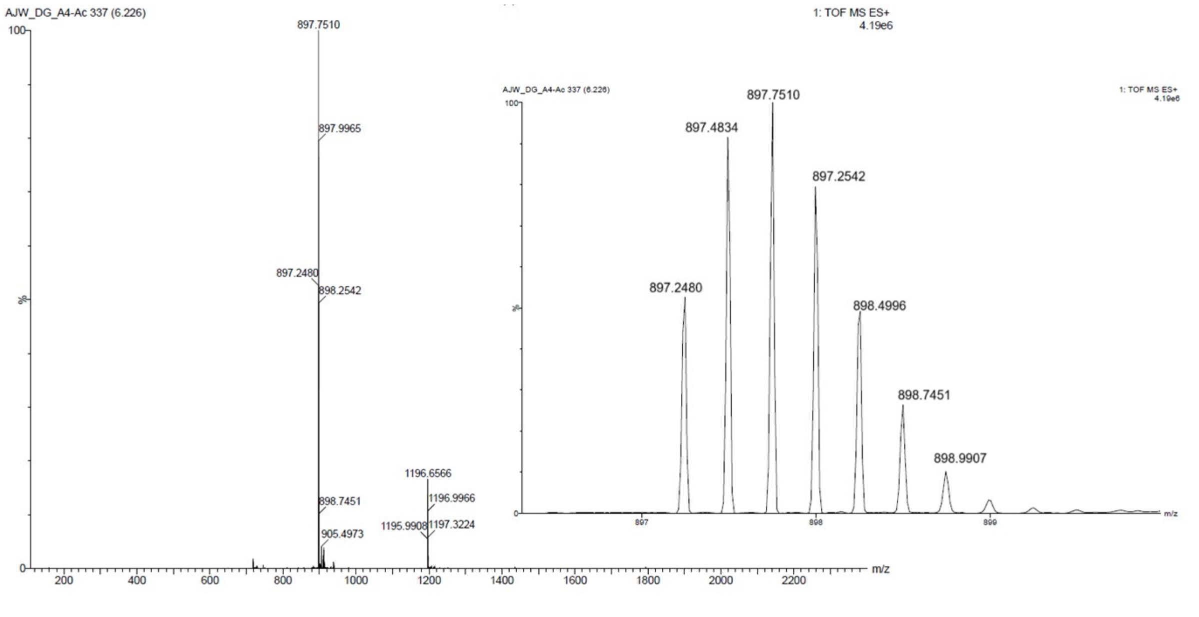


**HR-QToF(ESI+)MS analysis of purified Ac-CC-Di-p53-4.**

**FAM-Ahx-CC-Di-p53-4**

**Sequence:**

FAM-Ahx- GEIAALKQEIAALKFENAWLKLEIEALKQGYG -$CONH_2$

HPLC (λ = 214 nm) tR = 12.421 min. HR-QToF (ESI) m/z calc. for **$C_{191}H_{284}N_{42}O_{53}$**: $[M+3H]^{3+}$: Calc: 1339.0346, Found: 1339.0363; $[M+4H]^{4+}$: Calc: 1004.5277, Found: 1004.5252. $[M+5H]^{5+}$: Calc: 803.8237, Found: 803.8241.

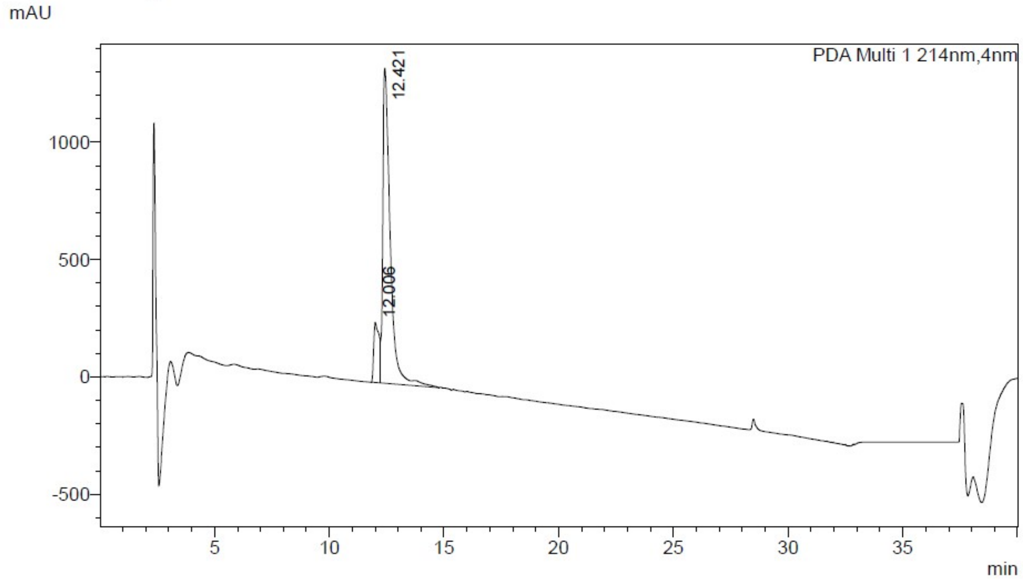


**Analytical HPLC trace at λ= 214 nm of purified FAM-Ahx--CC-p53-4.**

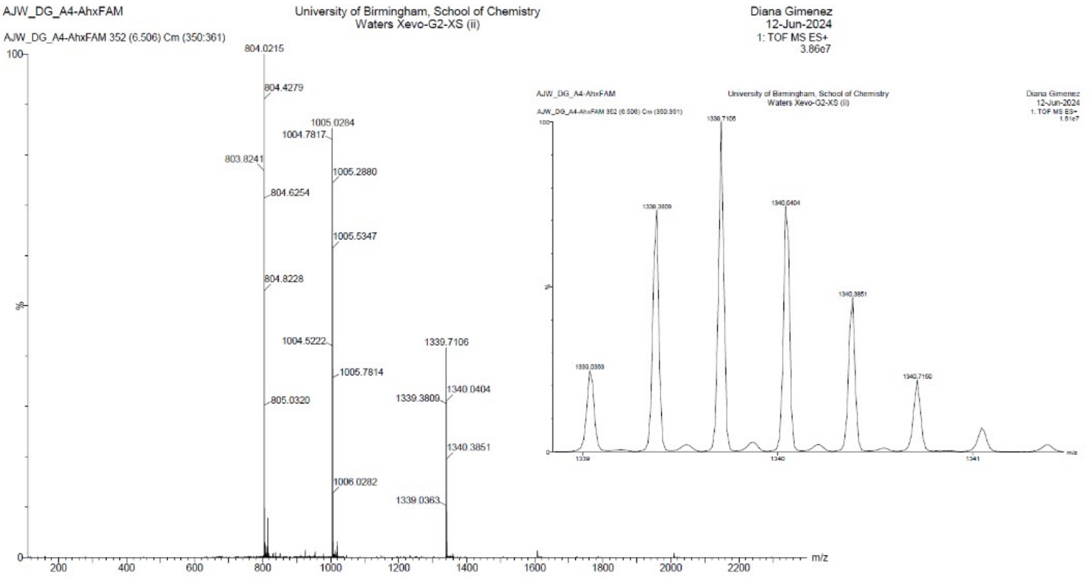


**HR-QToF(ESI+)MS analysis of purified FAM-Ahx--CC-Di-p53-4.**

## Ac-CC-Di-p53-5

**Sequence:**

Ac- GEIAALKQEIAALKFENLWLKLEIAALKQGYG-$CONH_2$

HPLC (λ = 214 nm) tR = 4.710 min. HR-QToF (ESI) m/z calc. for $\mathbf{C_{167}H_{269}N_{41}O_{45}}$: $[M+3H]^{3+}/[2M+6H]^{6+}$: Calc: 1190.6747, Found: 1190.6635; $[M+4H]^{4+}$: Calc: 893.2578, Found: 893.2570.

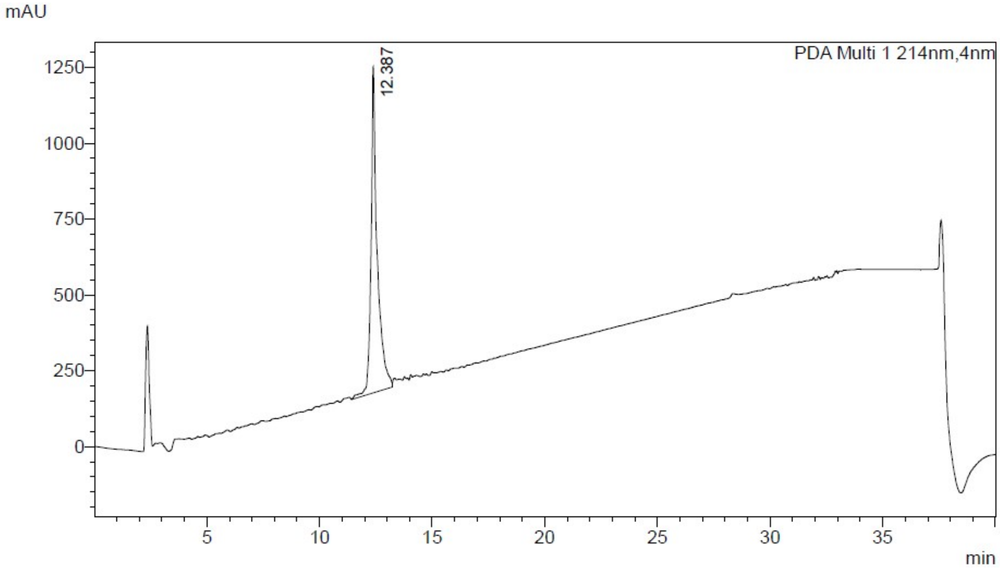


**Analytical HPLC trace at λ= 214 nm of purified Ac-CC-Di-p53-5.**

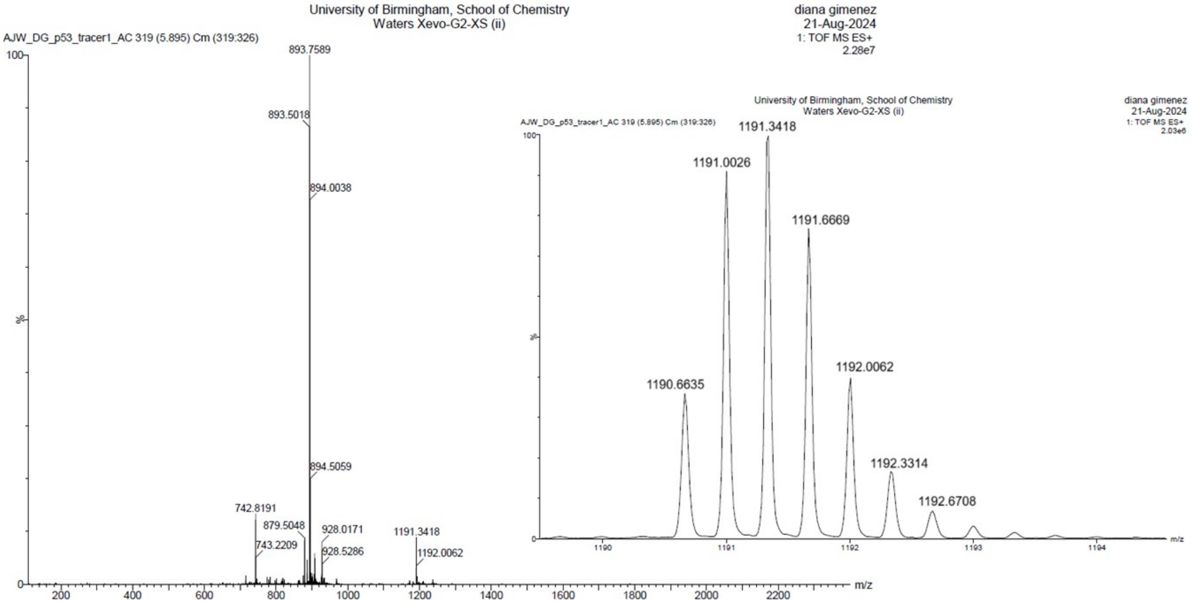


**HR-QToF(ESI+)MS analysis of purified Ac-CC-Di-p53-5.**

**FAM-Ahx-CC-Di-p53-5**

**Sequence:**

FAM-Ahx-GEIAALKQEIAALKFENLWLKLEIAALKQGYG-CONH$_2$

HPLC (λ = 214 nm) tR = 12.835 min. HR-QToF (ESI) m/z calc. for **$C_{192}H_{288}N_{42}O_{51}$**: $[M+3H]^{3+}$/$[2M+6H]^{6+}$: Calc: 1333.7151, Found: 1333.7012; $[M+4H]^{4+}$/$[2M+8H]^{8+}$: Calc: 1000.5381, Found: 1000.5164; $[M+5H]^{5+}$/$[2M+10H]^{10+}$: Calc: 800.6319.5381, Found: 893.6185

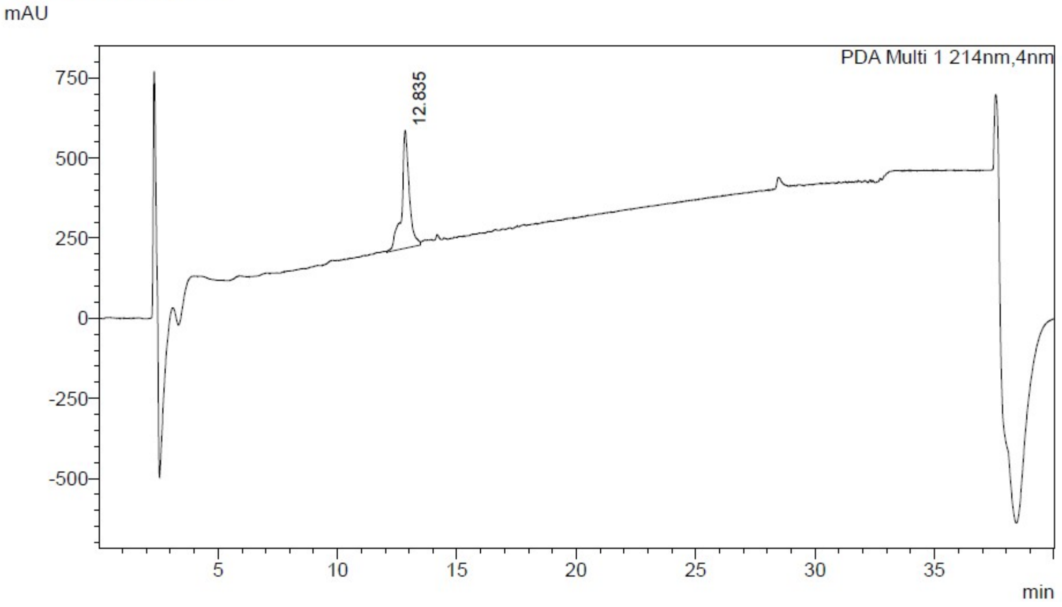


**Analytical HPLC trace at λ= 214 nm of purified FAM-Ahx-CC-Di-p53-5.**

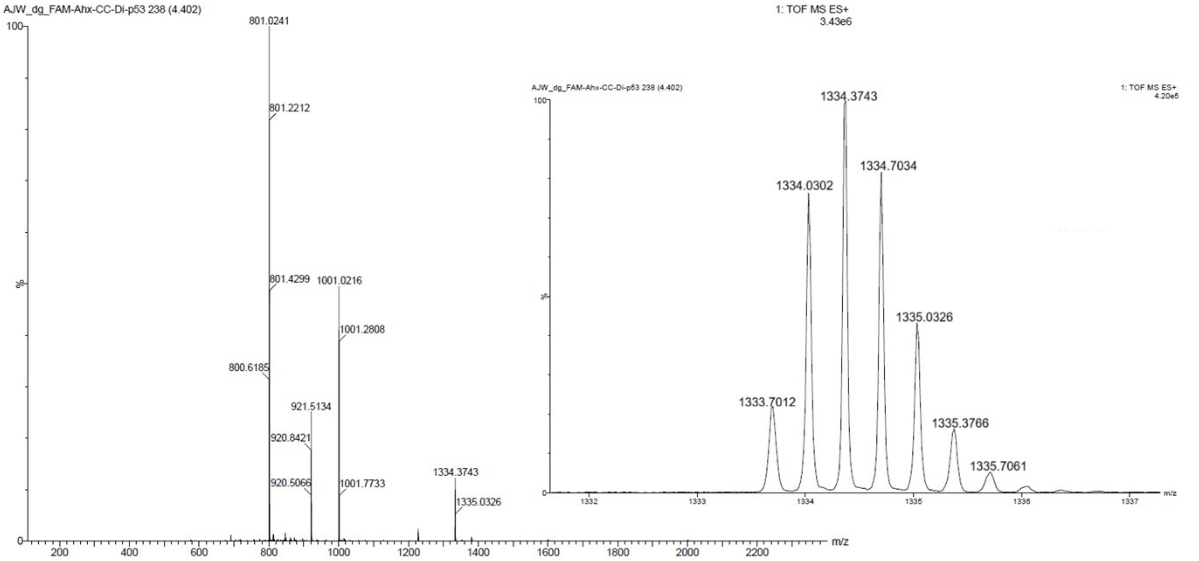


**HR-QToF(ESI+)MS analysis of purified FAM-Ahx-CC-Di-p53-5.**

**Ac-CC-Di-p53-6**

**Sequence:**

Ac- ALKQEIAALKFEIYWLKLENAALKQEIA -$CONH_2$

HPLC (λ = 214 nm) tR = 4.710 min. HR-QToF (ESI) m/z calc. for $\mathbf{C_{155}H_{249}N_{37}O_{41}}$: $[M+2H]^{2+}$ Calc: 1643.4341, Found: 1643.4271; $[M+3H]^{3+}$: Calc: 1095.9585, Found: 1095.9598. $[M+4H]^{4+}$: Calc: 822.2207, Found:822.2245.

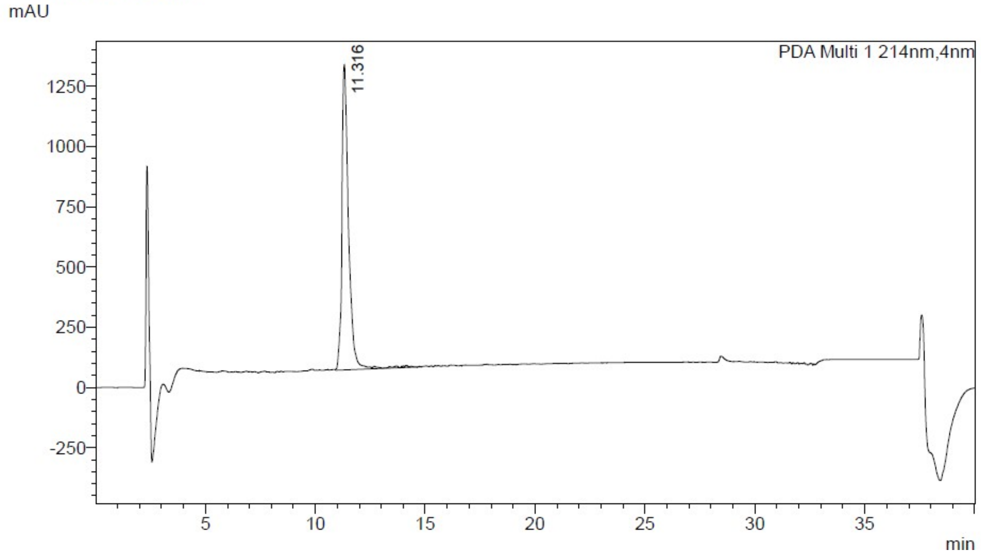


**Analytical HPLC trace at λ= 214 nm of purified Ac-CC-Di-p53-6**

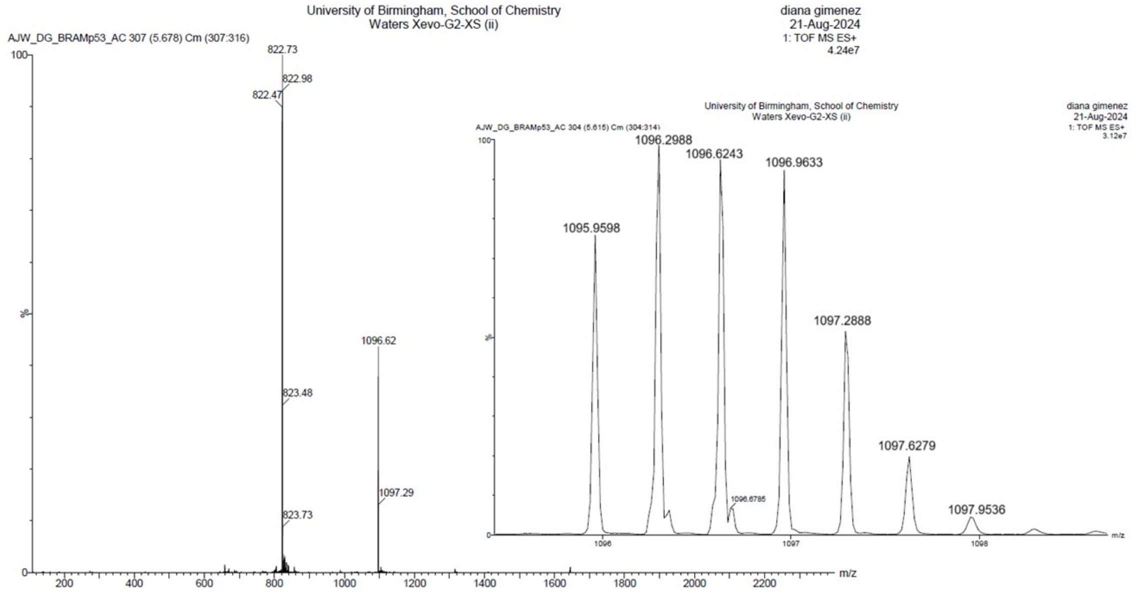


**HR-QToF(ESI+)MS analysis of purified Ac-CC-Di-p53-6.**

**FAM-Ahx-CC-Di-p53-6**

**Sequence:**

FAM-Ahx- ALKQEIAALKFEIYWLKLENAALKQEIA -$CONH_2$

HPLC (λ = 214 nm) tR = 4.710 min. HR-QToF (ESI) m/z calc. for $\mathbf{C_{180}H_{268}N_{38}O_{47}}$: $[M+3H]^{3+}$: Calc: 1238.9989, Found: 1238.9958; $[M+4H]^{4+}$: Calc: 929.5010, Found:929.4971; $[M+5H]^{5+}$: Calc: 743.8023, Found:743.7947.

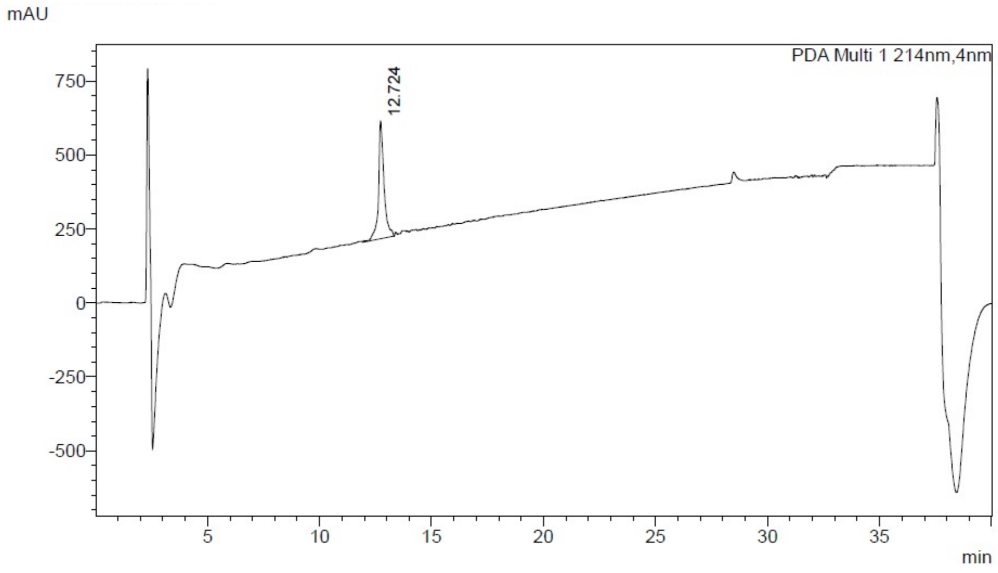


**Analytical HPLC trace at λ= 214 nm of purified FAM-Ahx-CC-Di-p53-6.**

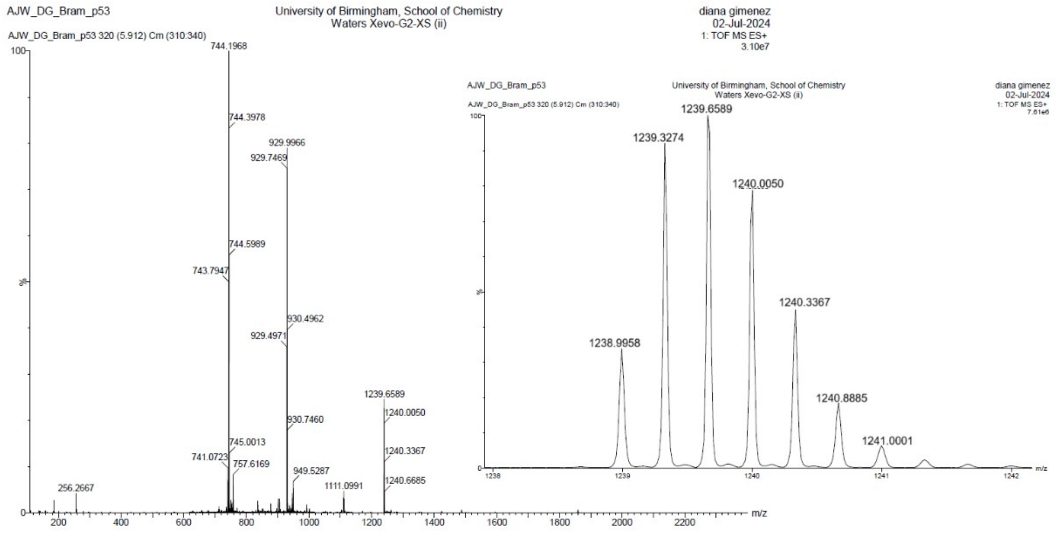


**HR-QToF(ESI+)MS analysis of purified FAM-Ahx-CC-Di-p53-6.**

**Ac-p53-Opt**

**Sequence:**

Ac- RFMDYWEGL-$CONH_2$

HPLC (λ = 214 nm) tR = 12.154 min. HR-QToF (ESI) m/z calc. for $\mathbf{C_{59}H_{80}N_{14}O_{15}S}$: $[M+H]^+$: Calc: 1257.5721, Found: 1257.5720; $[M+2H]^{2+}$: Calc: 629.2897, Found: 629.2951.

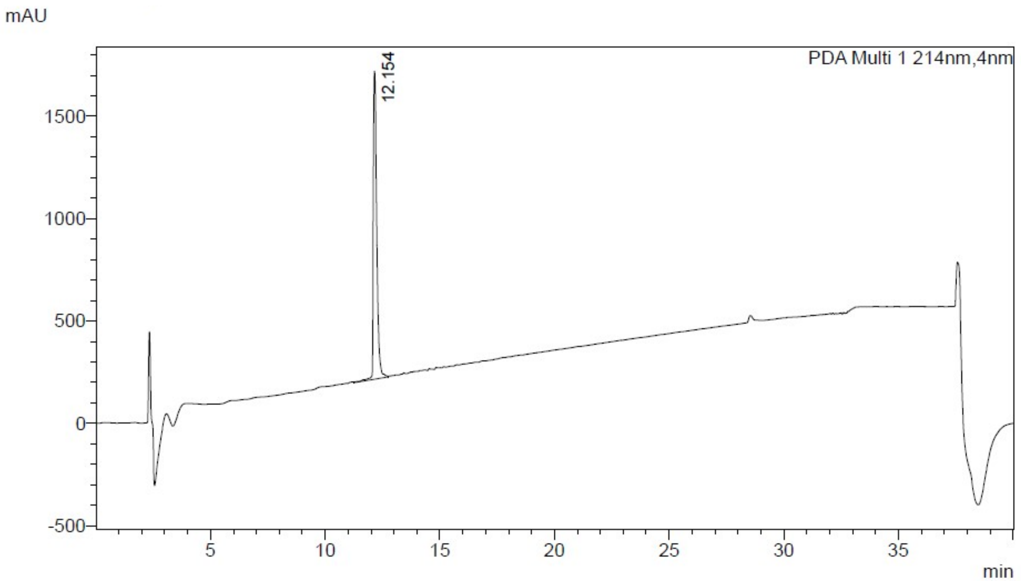


**Analytical HPLC trace at λ= 214 nm of purified Ac-p53-Opt.**

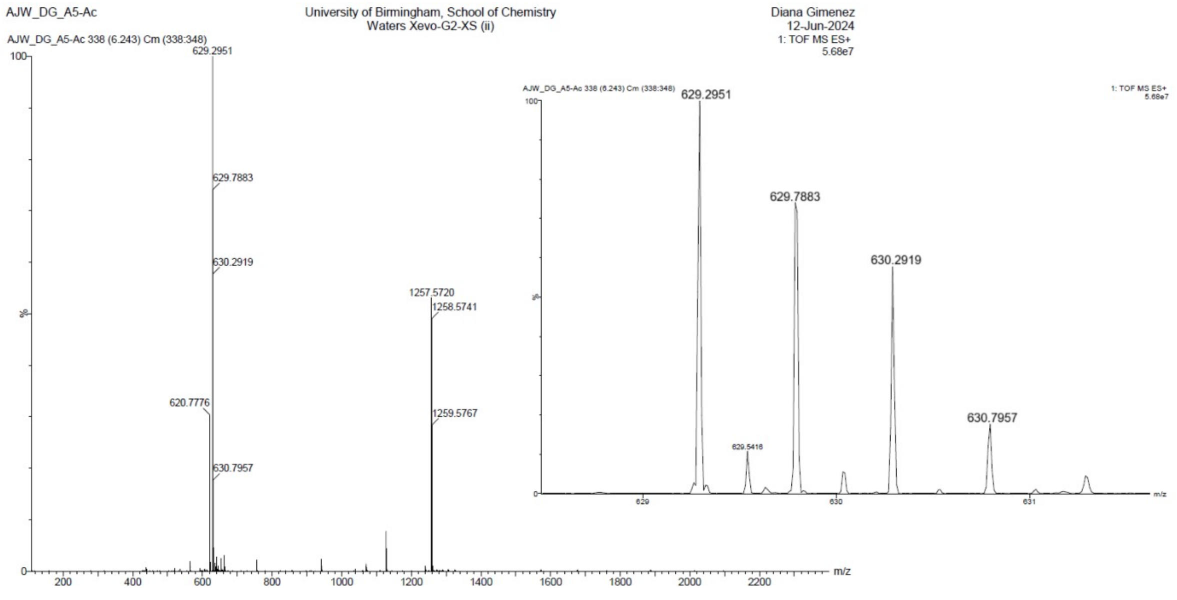


**HR-QToF(ESI+)MS analysis of purified Ac-p53-Opt.**

**FAM-Ahx-p53-Opt**

**Sequence:**

FAM-Ahx- RFMDYWEGL-CONH$_2$

HPLC (λ = 214 nm) tR = 13.854 min. HR-QToF (ESI) m/z calc. for **$C_{84}H_{99}N_{15}O_{21}S$**: $[M+H]^{+}$: Calc: 1686.6933, Found: 1686.7053; $[2M+3H]^{3+}$: Calc: 1124.7980, Found: 1124.7904; $[M+3H]^{3+}$; $[M+2H]^{2+}$: Calc: 843.8503, Found: 843.8526; $[M+3H]^{3+}$: Calc: 562.9026, Found: 562.8970.

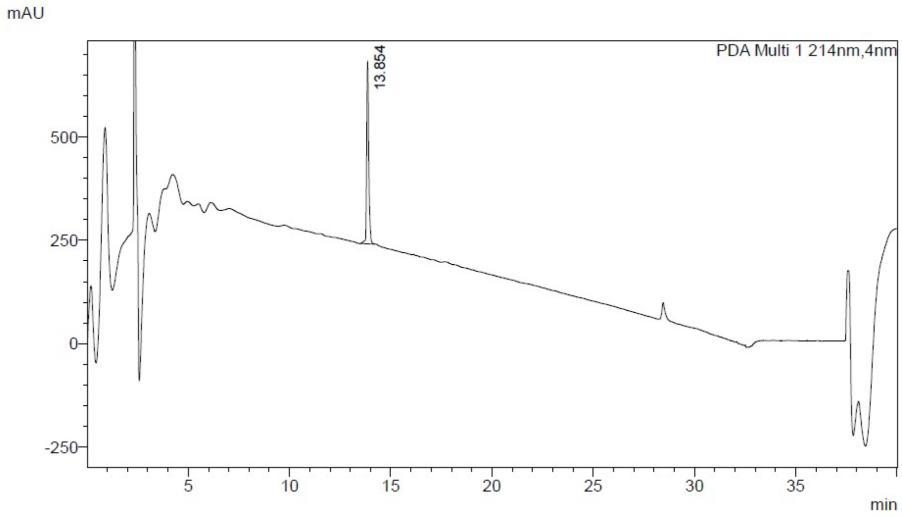


**Analytical HPLC trace at λ= 214 nm of purified FAM-Ahx-p53-Opt.**

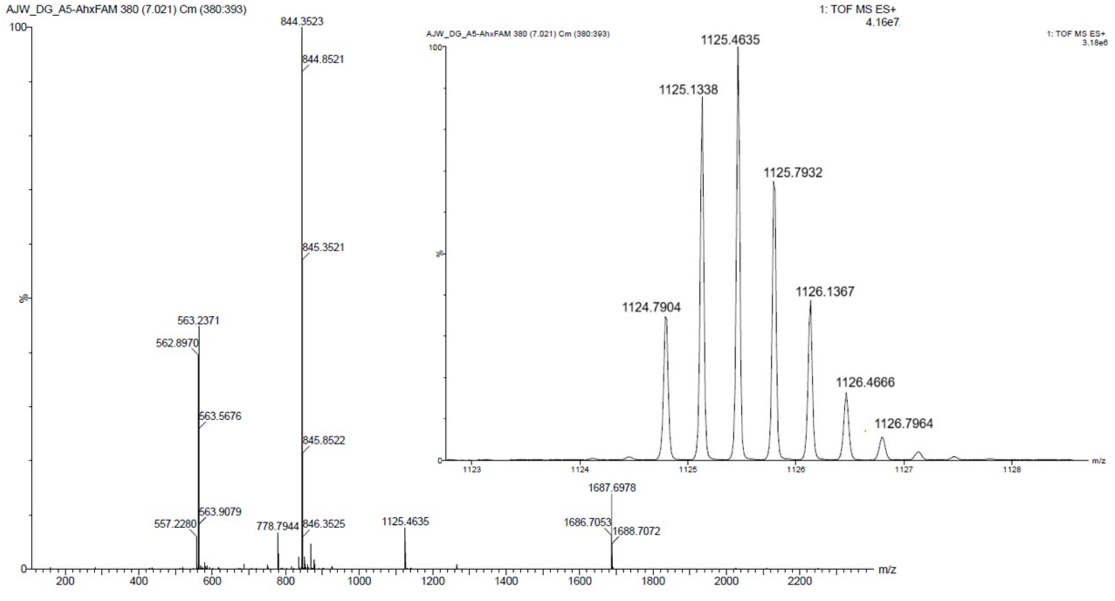


**HR-QToF(ESI+)MS analysis of purified FAM-Ahx-p53-Opt.**

**References**


1. A. Czarna, G. M. Popowicz, A. Pecak, S. Wolf, G. Dubin and T. A. Holak, *Cell Cycle*, 2009, **8**, 1176-1184.
2. C. Li, M. Pazgier, M. Liu, W.-Y. Lu and W. Lu, *Angew. Chem. Int. Ed.*, 2009, **48**, 8712-8715.
3. J. Phan, Z. Li, A. Kasprzak, B. Li, S. Sebti, W. Guida, E. Schönbrunn and J. Chen, *J. Biol. Chem.*, 2010, **285**, 2174-2183.
4. C. Li, M. Pazgier, C. Li, W. Yuan, M. Liu, G. Wei, W.-Y. Lu and W. Lu, *J. Mol. Biol.*, 2010, **398**, 200-213.
5. B. Anil, C. Riedinger, J. A. Endicott and M. E. M. Noble, *Acta. Crystallogr. D*, 2013, **69**, 1358-1366.
6. C. W. Wood, A. A. Ibarra, G. J. Bartlett, A. J. Wilson, D. N. Woolfson and R. B. Sessions, *Bioinformatics*, 2020, **36**, 2917-2919.
7. A. I. Green, F. Hobor, C. P. Tinworth, S. Warriner, A. J. Wilson and A. Nelson, *Chem. Eur. J.*, 2020, **26**, 10682-10689.
8. A. M. Acevedo-Jake, B. Mylemans, D. F. Kay, P. Zhang, B. Korona, G. G. Rhys, A. C. Leney, D. T. Huang, T. A. Edwards, L. Itzhaki, D. N. Woolfson and A. J. Wilson, *ACS Chem. Biol.*, 2025, **20**, 1309-1318.
